# Transcription Factor CTIP1/ BCL11A Regulates Epidermal Differentiation and Lipid Metabolism During Skin Development

**DOI:** 10.1038/s41598-017-13347-7

**Published:** 2017-10-18

**Authors:** Shan Li, Amy Teegarden, Emily M. Bauer, Jaewoo Choi, Nadia Messaddeq, David A. Hendrix, Gitali Ganguli-Indra, Mark Leid, Arup K. Indra

**Affiliations:** 1 Department of Pharmaceutical Sciences, College of Pharmacy, OSU, Corvallis, Oregon, 97331 USA; 2 Department of Biochemistry and Biophysics, OSU, Corvallis, Oregon, 97331 USA; 3 Linus Pauling Science Center, OSU, Corvallis, Oregon, 97331 USA; 4 Institut de Génétique et de Biologie Moléculaire et Cellulaire, Department of Functional Genomics, Inserm U596 and CNRS UMR 7104, Illkirch-Graffenstaden, 67400 France; 5 Knight Cancer Institute, Portland, Oregon, 97239 USA; 6 Department of Integrative Biosciences, Oregon Health & Science University (OHSU), Portland, Oregon, 97239 USA; 7 Departments of Dermatology, Oregon Health & Science University (OHSU), Portland, Oregon, 97239 USA

## Abstract

The epidermal permeability barrier (EPB) prevents organisms from dehydration and infection. The transcriptional regulation of EPB development is poorly understood. We demonstrate here that transcription factor COUP-TF-interacting protein 1 (CTIP1/BCL11A; hereafter CTIP1) is highly expressed in the developing murine epidermis. Germline deletion of *Ctip1* (*Ctip1* ^−/−^) results in EPB defects accompanied by compromised epidermal differentiation, drastic reduction in profilaggrin processing, reduced lamellar bodies in granular layers and significantly altered lipid composition. Transcriptional profiling of *Ctip1* ^−/−^ embryonic skin identified altered expression of genes encoding lipid-metabolism enzymes, skin barrier-associated transcription factors and junctional proteins. CTIP1 was observed to interact with genomic elements within the regulatory region of the gene encoding the differentiation-associated gene, *Fos-related antigen2 (Fosl2*) and lipid-metabolism-related gene, *Fatty acid elongase 4 (Elvol4*), and the expression of both was altered in *Ctip1* ^−/−^ mice. CTIP1 appears to play a role in EPB establishment of via direct or indirect regulation of a subset of genes encoding proteins involved in epidermal differentiation and lipid metabolism. These results identify potential, CTIP1-regulated avenues for treatment of skin disorders involving EBP defects.

## Introduction

Mammalian skin, the largest organ in the body, is composed of epidermis and the underlying dermis^1–3^. The epidermis serves as a barrier to protect the body against both dehydration and external toxic and pathogenic agents^2–4^. During embryonic development, the epidermis forms from the primitive ectoderm, developing into a multilayered stratified epithelium consisting of basal, spinous, granular and the outermost cornified cell layers^2,3^. The structural integrity of the epidermis is dependent on the proper and tightly regulated differentiation of keratinocytes, the most abundant cell type in the epidermis^1–3,5^.

Skin barrier defects are known to contribute to the pathogenesis of several skin diseases [e.g. atopic dermatitis (AD), ichthyosis and psoriasis]^6–9^. The disruption of epidermal differentiation, alteration of lipid composition and cell-cell junction formation have been reported to affect skin barrier functions^3,6,10^. The stratum corneum (SC), comprised of lipid-embedded corneocytes and a lipid-rich extracellular matrix, plays a key role in formation and maintenance of the skin permeability barrier^11^. Alteration of SC lipid composition leads to impaired skin barrier functions and results in an increase in trans-epidermal water loss (TEWL). Major lipid constituents of the SC include ceramides (CERs), free fatty acids (FFAs), cholesterol, and triglycerides (TGs)^12^. CERs are the most important epidermal sphingolipids and most abundant lipid types (50% by weight). A large number of CER subclasses exists with a wide variation in chain length distribution^13^. Four major pathways of ceramide biosynthesis and metabolism have been reported in skin: (1) *de novo* synthesis pathway; (2) sphingomyelinase pathway; (3) salvage pathway and (4) exogenous ceramide recycling pathway. Different enzymes of the sphingolipid metabolic pathways, such as serine palmitoyltransferase, ceramide synthase, glucosylceramide synthase, acid beta-glucosidase, sphingomyelin synthase, and fatty acid elongase, play important roles in epidermal signaling^14^. Furthermore, cell-cell junctions, such as desmosomes, gap junctions and tight junctions (TJs), are also critical for proper skin barrier functions^15–17^.

A variety of transcription factors (TFs) and signaling pathways are crucial for establishing the functional epidermal permeability barrier (EPB). For example, the p53 family member p63 is a master regulator for epidermal proliferation and differentiation^18^ and barrier deficiencies in *Gata3* ^−/−^, *Klf4* ^−/−^ and *Fosl2* ^−/−^ mice^19,20^^,^^21^. In addition, the EGFR-NOTCH, Shh and Wnt signaling pathways have been implicated in control of keratinocyte differentiation^22–24^.

Chicken ovalbumin upstream promoter transcription factor (COUP-TF)-interacting protein 1 [(CTIP1; also known as B-cell CLL/lymphoma 11 A (BCL11A)] is a zinc finger transcriptional factor that shares many functional properties with its homolog CTIP2/BCL11B. The two centrally located zinc fingers, 3 and 4 in CTIP1/BCL11A and 2 and 3 in CTIP2/BCL11B, exhibit 94% identity^25,26^. CTIP1 is expressed in the murine central nervous system (CNS) during embryogenesis^27,28^, and in murine hematopoietic systems during development and in adulthood^29–31^. CTIP1 is essential for post-natal development and normal lymphopoiesis^29,32^. Defects in cell-cycle and multi-lineage differentiation in *Ctip1-deficient* HSCs have been revealed by single-cell analyses^30^. A recent study identified CTIP1 as a critical regulator of normal mammary epithelial development^33^. We have shown that CTIP2 is highly expressed in skin and plays a significant role in barrier establishment, lipid metabolism and epidermal homeostasis, as well as in hair follicle morphogenesis and hair cycling^14,34,35^. However, the expression pattern of CTIP1 in skin and its functions in EPB formation, epidermal proliferation and differentiation during development are unknown.

Here, we found that CTIP1 was highly expressed in the developing murine skin. Germline deletion of *Ctip1* resulted in EPB defects accompanied by compromised skin differentiation and altered skin lipid composition. Moreover, altered expression of genes encoding lipid-metabolism enzymes, skin barrier-associated TFs, and junctional proteins (JPs) was observed in *Ctip1* ^−/−^ embryonic skin. Finally, we demonstrated that CTIP1 interacts with the promoter regions of *Fosl2* and *Elvol4*, genes that are associated with epidermal differentiation and lipid metabolism, respectively. Taken together, this study describes a new *in vivo* role of CTIP1 in establishing the epidermal permeability barrier during murine skin morphogenesis.

## Results

### CTIP1 is expressed in developing and adult mouse skin

**Figure 1:**
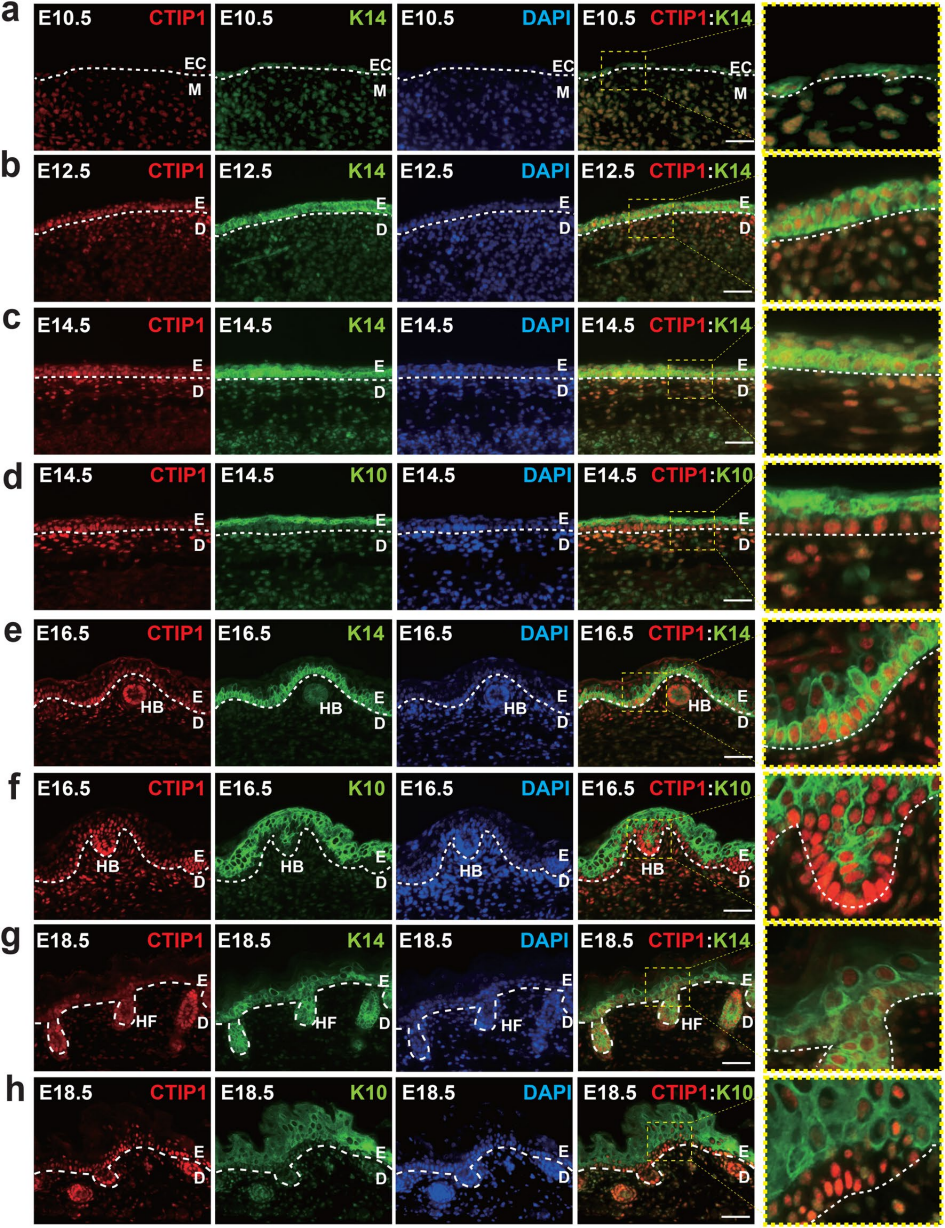
Expression of CTIP1 in developing mouse skin. (**a**,**b**,**c**,**e**,**g**) IHC staining for co-expression of CTIP1 (red) and Keratin K14 (green) at indicated days (d,f,h) Co-expression of CTIP1 (red) and K10 (green) at indicated days. Keratin 14 (K14) and Keratin 10 (K10) staining marks the basal and suprabasal cell layers, respectively. All sections were counterstained with DAPI (blue) to stain the nuclei. EC, ectoderm; M mesoderm; E, epidermis; D, dermis; HB, hair bulbs; HF, hair follicle. Scale bar = 50 μm.

Immunohistochemistry was performed at different stages of skin development using anti-CTIP1 antibody to characterize the expression pattern of CTIP1 during mouse skin morphogenesis. CTIP1 expression was detected in the single-layered ectoderm as early as embryonic day 10.5 (E10.5). CTIP1 co-localized with keratin 14 (K14) in the ectoderm E10.5 and E12.5 (Fig. 1a,b). At E14.5, CTIP1 was mainly and persistently expressed in the rapidly dividing basal cell layer (Fig. 1c). Expression of CTIP1 was also detected in some cells in the suprabasal layers, co-localized with early differentiation marker keratin 10 (K10) (Fig. 1d). At E16.5 and E18.5, high levels of CTIP1 expression were observed and co-localized with K14 in the basal layer of the epidermis, as well as in the hair bulbs and follicles. In the suprabasal layers, few CTIP1 positive cells were observed at E16.5 and E18.5 (Fig. 1e–h). Some of the dermal cells were found to express CTIP1 at early stages from E10.5 (Fig. 1a–f), but expression decreased at later stages and more restricted to the epidermis ~E18.5, when the fully stratified and differentiated epidermis is formed (Fig. 1g,h)^2,3^. We found a similar expression pattern in the skin of 8-week-old adult mice, with majority of cells in the basal layer, hair follicles and some cells in the suprabasal layers expressing CTIP1 (Figure S1a and b). However, immunoblot analysis revealed that expression of CTIP1 in the E18.5 embryonic skin was higher than in the adult skin (Figure S1c and d). These observations suggest a role for CTIP1 in murine skin, particularly during embryonic development, and form the cornerstone of the studies performed hereafter.

### Disruption of skin permeability barrier function in *Ctip1*^−/−^ embryos during development

**Figure 2:**
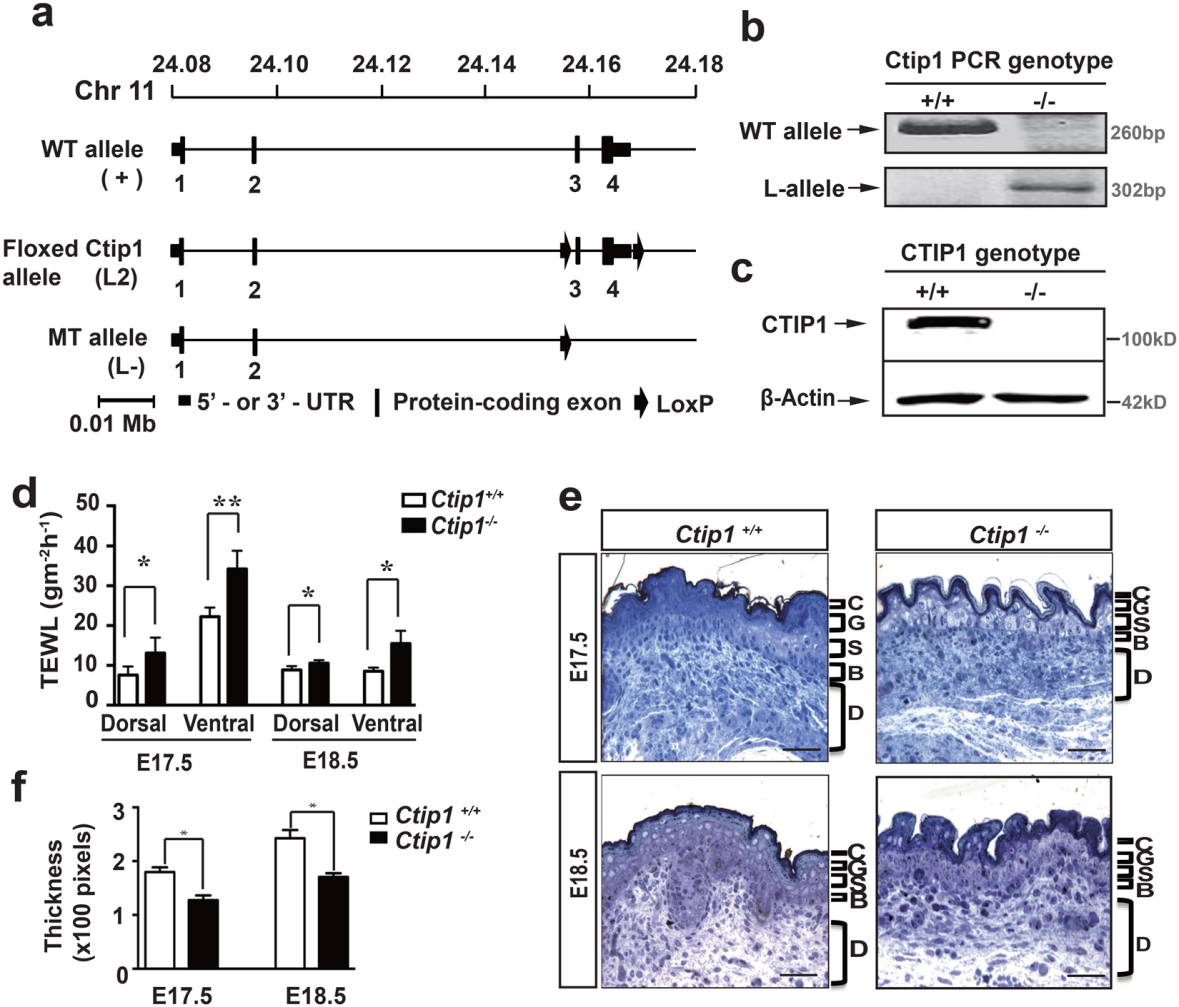
Generation and characterization of *Ctip1* ^−/−^ mice. (**a**) Schematic representation of *Ctip1* wild-type ( + ), floxed (L2) and mutant (L-) alleles. Exons (1–4) are indicated by black boxes. (**b**) PCR analysis of *Ctip1* DNA fragments, and (**c**) Immunoblot analysis of protein extracts from *Ctip1* ^+/+^ and *Ctip1* ^−/−^ embryonic skin using anti-CTIP1 monoclonal antibody. β-actin was used as a loading control. (**d**) TEWL measurement of wild-type and *Ctip1* ^−/−^ embryos on dorsal and ventral skin using a tewameter at E17.5 and E18.5. (**e**) Histology of toluidine blue stained ventral skin biopsies (2-μm thick sections) from *Ctip1* ^+/+^ and *Ctip1* ^−/−^ embryos at E17.5 and E18.5. (**f**) Quantitative histomorphometry of *Ctip1* ^−/−^ phenotype. C, cornified; G, granular; S, spinous; B, basal layers, D, dermis. *P < 0.05, **P < 0.01.

To determine the function of CTIP1 in epidermal morphogenesis, we generated *Ctip1-null* mice and analyzed their skin. The Cre-LoxP system was used to flox exons 3 and 4 of the *Ctip1* locus, which together encode ~75% of the total protein-coding sequence of *Ctip1* including the DNA binding domain (Fig. 2a). Deletion of *Ctip1* was confirmed by PCR (Fig. 2b), immunoblotting, and IHC (Fig. 2c, Figure S2a). Heterozygous mutant mice (*Ctip1* ^+/−^) were phenotypically indistinguishable from WT littermates. In contrast, the *Ctip1* ^−/−^ mice died shortly (~2 hours) after birth. As *Ctip2* ^−/−^ mice, a paralogue of *Ctip1*, also died shortly after birth and displayed skin barrier defects^35^, we examined EPB function in *Ctip1* ^−/−^ mice by the X-gal dye diffusion assay. At E17.5, the *Ctip1* ^−/−^ embryos exhibited positive blue staining on the ventral surface, whereas the WT littermates stained negative (Figure S2b). However, positive staining was not observed in mutants or WT embryos at E18.5 (Figure S2b). Because the dye diffusion assay is less precise than measurement of TEWL^36^, we measured TEWL in those embryos. A significant increase in water loss through dorsal and ventral skin of *Ctip1* ^−/−^ embryos was observed compared to that of WT controls at both E17.5 and E18.5 (Fig. 2d). Furthermore, histological analyses on 2μm thick semithin sections revealed that the epidermis of *Ctip1* ^−/−^ embryos was significantly thinner than WT embryos at both stages (Fig. 2e,f). These results demonstrate that *Ctip1* ^−/−^ embryos exhibit significant barrier defects and suggest an important role of CTIP1 in formation and establishment of EPB. Skin samples from E18.5 WT and *Ctip1* ^−/−^ embryos in all our subsequent studies.

### Altered epidermal differentiation in the skin of *Ctip1*^−/−^ embryos

**Figure 3:**
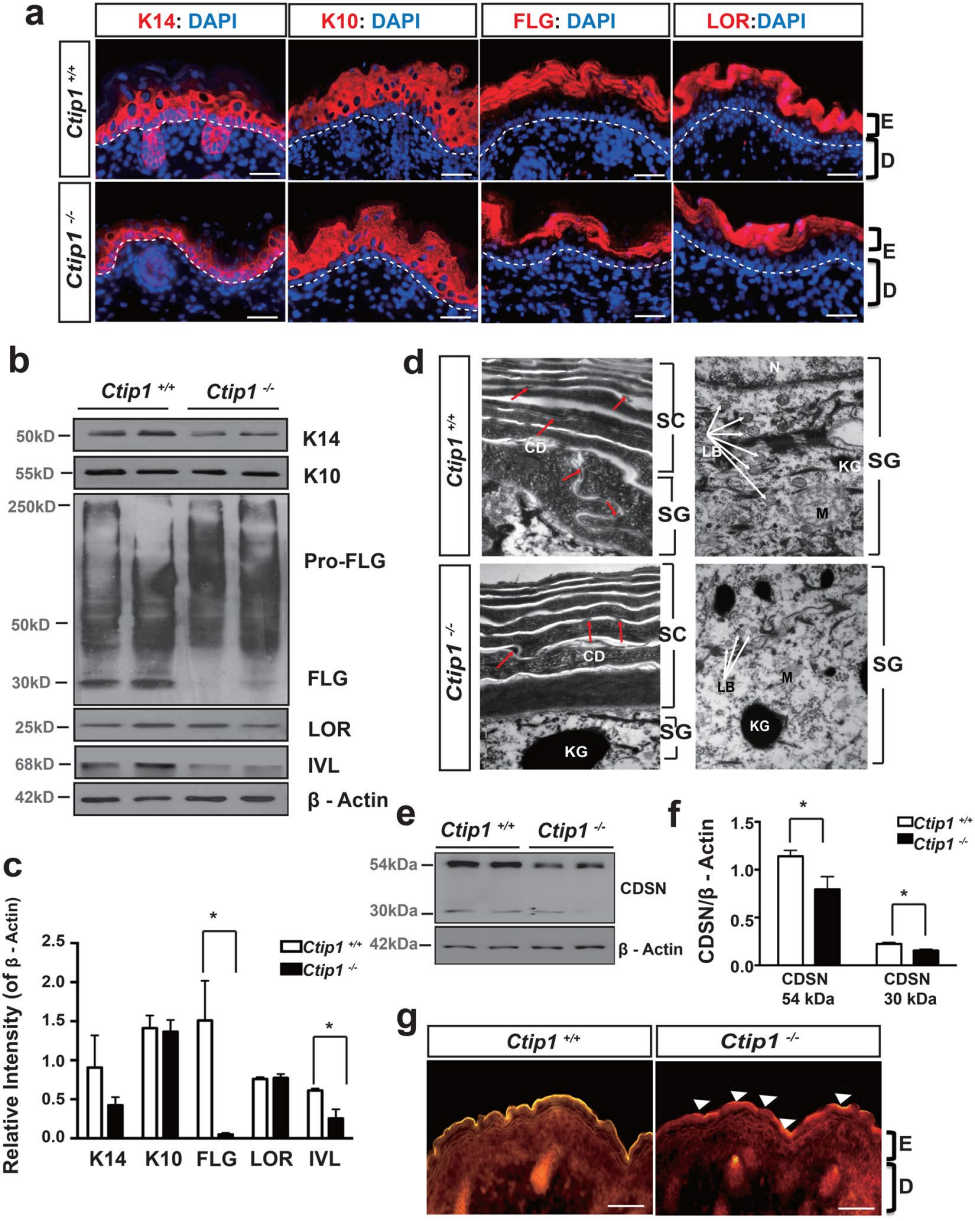
Altered epidermal differentiation and abnormal epidermal ultrastructures in *Ctip1* ^−/−^ embryos. (**a**) IHC of ventral skin with basal keratinocyte marker K14, early differentiation marker K10 and later differentiation markers loricrin (LOR) and filaggrin (FLG)^80^ in *Ctip1* ^+/+^ and *Ctip1* ^−/−^ embryos at E18.5. All 5μm-thick paraffin sections were counterstained with DAPI (blue). (**b**) Immunoblot of ventral skin extracts from *Ctip1* ^+/+^ and *Ctip1* ^−/−^ embryos at E18.5 using antibodies against K14, K10, FLG, LOR and involucrin (IVL). β-actin was used as a loading control. (**c**) Quantification of immunoblot, *P < 0.05. Full-length blots are presented in Supplementary Figure S7. (**d**) Transmission electron microscopy of ventral skin from *Ctip1* ^+/+^ and *Ctip1* ^−/−^ embryos at E18.5. White Arrows indicate lamellar bodies (LBs) in the granular layer of control and mutant embryos. Red arrows indicate corneodesmosomes. Scale bar = 1 μm. (**e**) Immunoblot of skin extracts from *Ctip1* ^+/+^ and *Ctip1* ^−/−^ embryos at E18.5 for Corneodesmosin (CDSN) using with anti-CDSN F28–27 mAb. β-actin was used as a loading control. (**f**) Quantification of immunoblot, *P < 0.05. (**g**) Nile Red staining of ventral skin sections from *Ctip1* ^+/+^ and *Ctip1* ^−/−^ embryos at E18.5. White Arrowheads indicate an absence of neutral lipids (stained golden yellow in the controls) on the skin surface of the mutant embryos. Scale bar = 50 μm. E, epidermis; D, dermis; SC, stratum corneum; SG, stratum granulosum; CD, corneodesmosomes; LB, lamellar bodies; KG, keratohyalin granules.

We hypothesized that the epidermal hypoplasia observed in the *Ctip1* ^−/−^ embryos could be due to alterations in epidermal proliferation and/or differentiation. To test that, we examined the expression of epidermal proliferation- and differentiation-markers, including K14, Ki67, and proliferating cell nuclear antigen (PCNA) (proliferation), K10 (early differentiation), involucrin (IVL) (differentiation), filaggrin (FLG) and loricrin (LOR) (late differentiation) in E18. 5 *Ctip1* ^−/−^ and *Ctip1* ^+/+^ embryos skin by IHC and immunoblot. The expression of K14 was modestly reduced in *Ctip1* ^−/−^ skin (Fig. 3a–c), although no significant differences in the percentage of Ki67^+^ basal cells (Figure S3a,b) or expression of PCNA were observed between WT and mutant skin (Figure S3c,d). Expression of IVL was decreased in *Ctip1* ^−/−^ skin compared to *Ctip1* ^+/+^ skin (Fig. 3b,c). IHC analysis also revealed that the distribution of FLG was altered in the granular layer of mutant skin (Fig. 3a). Immunoblot analysis further revealed that while pro-FLG was normally processed to FLG monomers in control epidermis, profilaggrin processing was dramatically disrupted and FLG monomers were greatly reduced in mutant skin (Fig. 3b and c). During epidermal terminal differentiation, profilaggrin is processed into mature FLG (FLG monomer), which is further broken down to release the natural moisturizing factor that is essential for epidermal hydration (Grishchuk *et al*., 2007). Caspase-14 is responsible for that FLG processing^37^, and it’s expression was also reduced in mutant skin (Figure S3e). No significant alterations in expression of K10 and LOR were observed in mutant skin. Collectively, results suggest that loss of CTIP1 leads to impaired terminal differentiation during skin development.

### Ultrastructural abnormalities and lipid distribution defects in the epidermis of *Ctip1*^−/−^ skin

Ultrastructural analyses were performed to characterize epidermal defects in *Ctip1* ^−/−^ skin. The numbers of desmosomes and corneodesmosomes (CDs) in the granular and cornified layers, as well as lamellar bodies in stratum granular (SG) layers, were significantly reduced in E18.5 mutant skin (Fig. 3d). Moreover, mutant CDs were much smaller in size than the control CDs (Fig. 3d). The protein level of corneodesmosin (CDSN), which is specific to CDs, was also significantly decreased in mutant skin compared to WT (Fig. 3e and f). The number and structure of keratin filaments in the SG layer of mutants was similar to that of WT mice. Lipid -rich lamellar bodies (LBs) were detected in the SG of *Ctip1* ^+/+^ embryonic skin. In contrast, the LBs in mutant skin were much smaller in size and were often empty and lacked their lipid contents (Fig. 3d). In order to test whether CTIP1 deficiency affected lipid distribution within the skin, Nile red staining was performed. We observed a golden-colored, continuous ribbon of neutral lipids along the top of the cornified layer of WT embryos, whereas those lipids were rarely detected in the mutant skin and were sometimes unevenly distributed on the surface of their epidermis (Fig. 3g). These results indicate that impaired barrier formation in *Ctip1* ^−/−^ embryos could be due to reduced LBs contents, altered distribution of lipids and impaired lipid metabolism in the developing skin.

### Altered composition of epidermal lipids in the skin of *Ctip1*^−/−^ embryos

**Figure 4:**
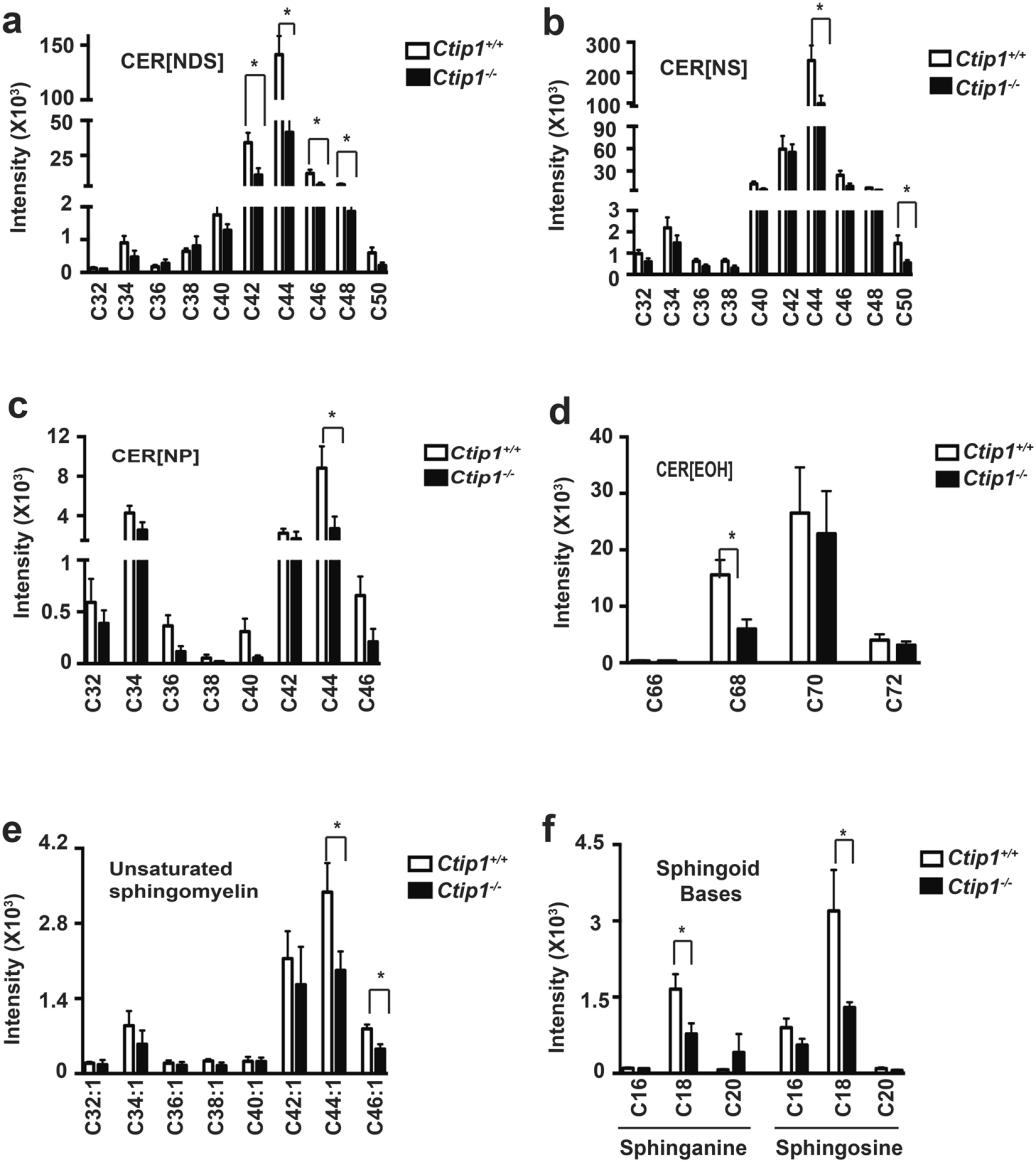
Altered profile of epidermal lipids in *Ctip1* mutant embryos (**a**–**f**) Significant alteration of different lipid species including (**a**–**d**) CER subclasses, (**e**) unsaturated sphingomyelin, and (**f**) sphingoid bases using LC-MS/MS in the skin of E18.5 *Ctip1* ^−/−^ embryos compared to the wild-type embryos. The X-axis defines the total carbon atom number, while the Y-axis indicates the absolute intensity (cps: count per second) of each lipid subclass. Each CER subclass is denoted by its sphingoid base and fatty acid chain and the total number of carbon atoms in CER is the number of carbon atoms in the fatty acid chain plus the number of carbon atoms in the sphingoid base. Sphinogid base abbreviations: DS, dihydrosphingosine; S, sphingosine; P, phytosphingosine; H, 6-hydroxy sphingosine. Acyl chain abbreviations: N, non-hydroxy fatty acid; EO, esterified w-hydroxy fatty acide. These abbreviations are combined to from subclass notations as shown in the figures: [NDS], [NS], [NP], [EOH]. Unsaturated sphingomyelin is expressed carbon number: double bonds number. Data are expressed as mean ± SEM (n = 8), *P < 0.05.

We hypothesized that CTIP1 plays a key role in regulating lipid homeostasis during skin development. We therefore performed lipidomic analyses to determine if epidermal lipid composition was altered in absence of CTIP1. All major SC lipids, including CERs, FFAs, cholesterol and TGs were detected by liquid chromatography-tandem mass spectrometry (*LC-MS/MS*). Seven of the 12 subclasses of CERs were identified in embryonic skin: CER[NDS], CER[NS], CER[NP], CER[NH], CER[AH], CER[EOS], and CER[EOH] (Figs 4, S4). CER[NS] and CER[NDS] were the most abundant in embryonic skin, while CER[NP], CER[NH], CER[AH], and very long-chain CER[EOS] and CER[EOH] were less abundant. We found specific CERs, belonging to four of the subclasses, were lower in mutant epidermis. These are long-chain fatty acids, such as CER [NDS] (42, 44, 46 and 48 carbons length) (Fig. 4a), CER[NS] (44 carbons length) (Fig. 4b), CER[NP] (44 carbons length) (Fig. 4c), as well as very long-chain CER[EOH] (68 carbons length) (Fig. 4d). The levels of CER[NH], CER[AH] and CER[EOS] were comparable between mutant and WT embryonic skin (Figure S4c).

The profile of saturated and unsaturated sphingomyelins, sphingosine, and sphinganine were also compared between WT and mutant embryonic skin. The long-chain sphingomyelins (44:1, 46:1 carbons length) were significantly reduced in mutant skin, while the composition of saturated sphingomyelins remained similar to WT (Fig. 4e and S 4d). In addition, the major free sphingoid bases, sphingosine and sphinganine (18 carbons long), were significantly reduced in mutant skin (Fig. 4f). The levels of both cholesterol and cholesterol 3-sulfate were comparable between the two groups (Figure S4e). Certain triglycerides (TGs), such as TG (52:2, 52:5) were significantly reduced in mutant skin compared to WT skin (Figure S4g). The above results confirm that epidermal lipid composition is significantly altered in *Ctip1* ^−/−^ embryos, possibly contributing to altered barrier functions during epidermal morphogenesis.

### Altered expression profile of genes implicated in lipid metabolism in *Ctip1*^−/−^ skin

**Figure 5:**
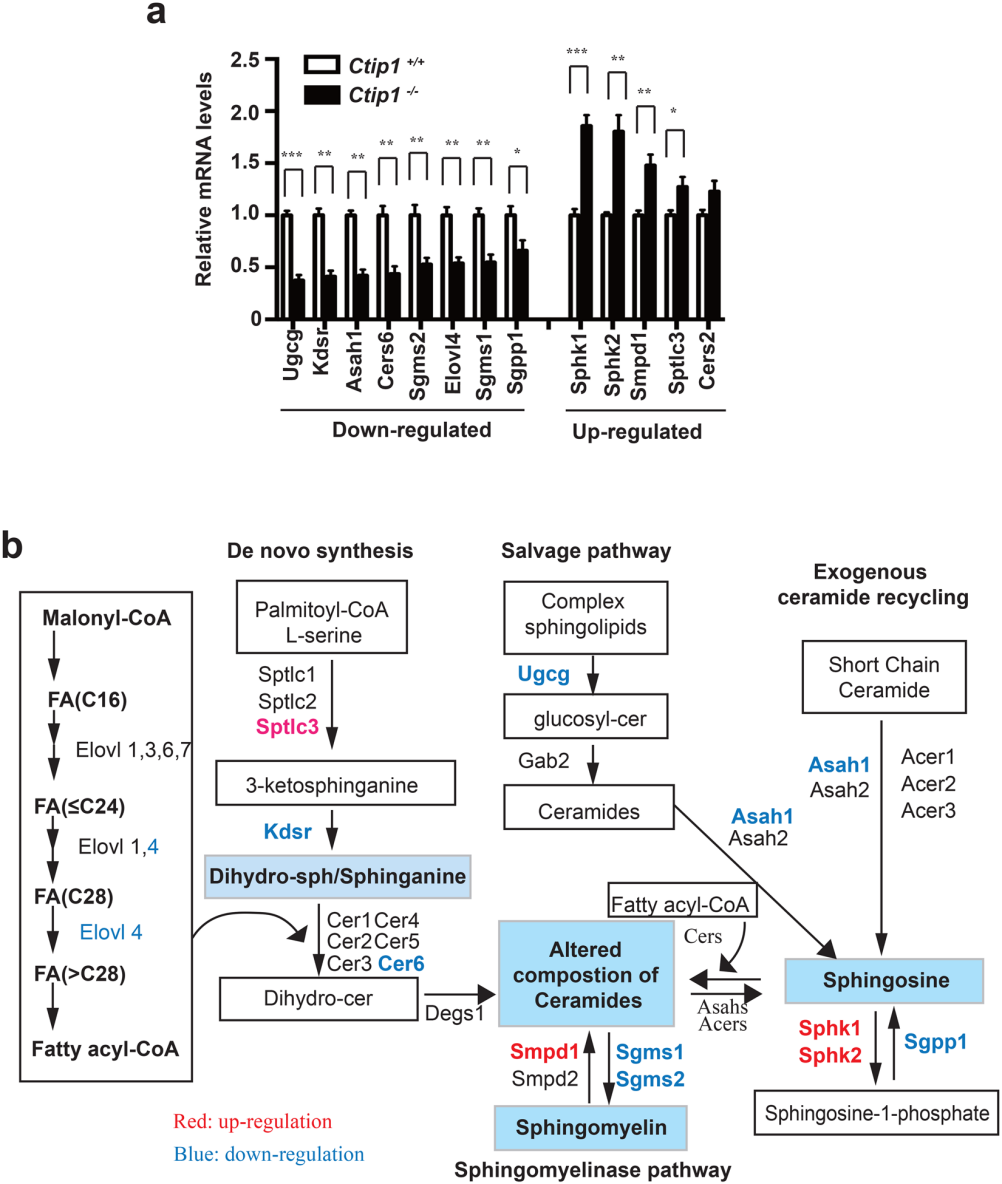
Differential expression of genes involved in sphingolipid metabolism in *Ctip1* mutant embryonic skin (**a**) Expression of several sphingolipid metabolism related genes was altered in *Ctip1* ^−/−^ embryonic skin based on RNA-seq (Figure S5b) by qRT-PCR. Data are expressed as mean ± SEM (n = 8 biological replicates), *P < 0.05, **P < 0.01. qRT-PCR for all genes were performed in triplicates. (**b**) Schematic representation of differentially expressed skin lipid-metabolizing genes in the *Ctip1* ^−/−^ embryos that are involved in ceramide biosynthesis pathways.

Altered lipid composition in *Ctip1* ^−/−^ embryos may be due to impaired expression of genes encoding proteins involved in lipid biosynthesis and/or processing, which was verified by RNA-seq analyses followed by qRT-PCR validation. RNA-seq analysis revealed that out of 38,052 genes, 4439 (11.7%) were differentially expressed in *Ctip1* ^−/−^ embryonic skin (FDR, p < 0.05). Among the significantly altered genes, 2233 were down-regulated and 2206 were up-regulated (Figure S5a). Based on RNA-seq analysis, we found a subset of genes involved in sphingolipid biosynthesis and metabolism pathways were either significantly up- or down-regulated in the mutant skin (Figure S5b). In particular, qRT-PCR validation results showed that expression of N-acylsphingosine amidohydrolase (*Asah1*), glucosylceramide synthase (*Ugcg*), 3-ketodihydrosphingosine reductase (*Kdsr*), CER synthase 6 (*Cers6*), *Elovl4*, and sphingomyelin synthase 1 and 2 (*Sgms1 and Sgms2*), were significantly decreased in mutant skin (Fig. 5a). In contrast, expression of sphingosine kinase 1 and 2 (*Sphk1* and *Sphk2*), sphingomyelinase 1 (*Smpd1*), and palmitoyltransferase (*Sptlc3*) was significantly increased in mutant skin (Fig. 5a). These differentially expressed genes, which encode lipid-metabolizing enzymes in the ceramide biosynthesis pathways (Figure 5b)^14^, indicate that CTIP1 deficiency interferes with epidermal lipid metabolism.

### Altered expression of genes encoding transcription factors (TFs) and junctional proteins (JPs) in *Ctip1*^−/−^ skin

**Figure 6:**
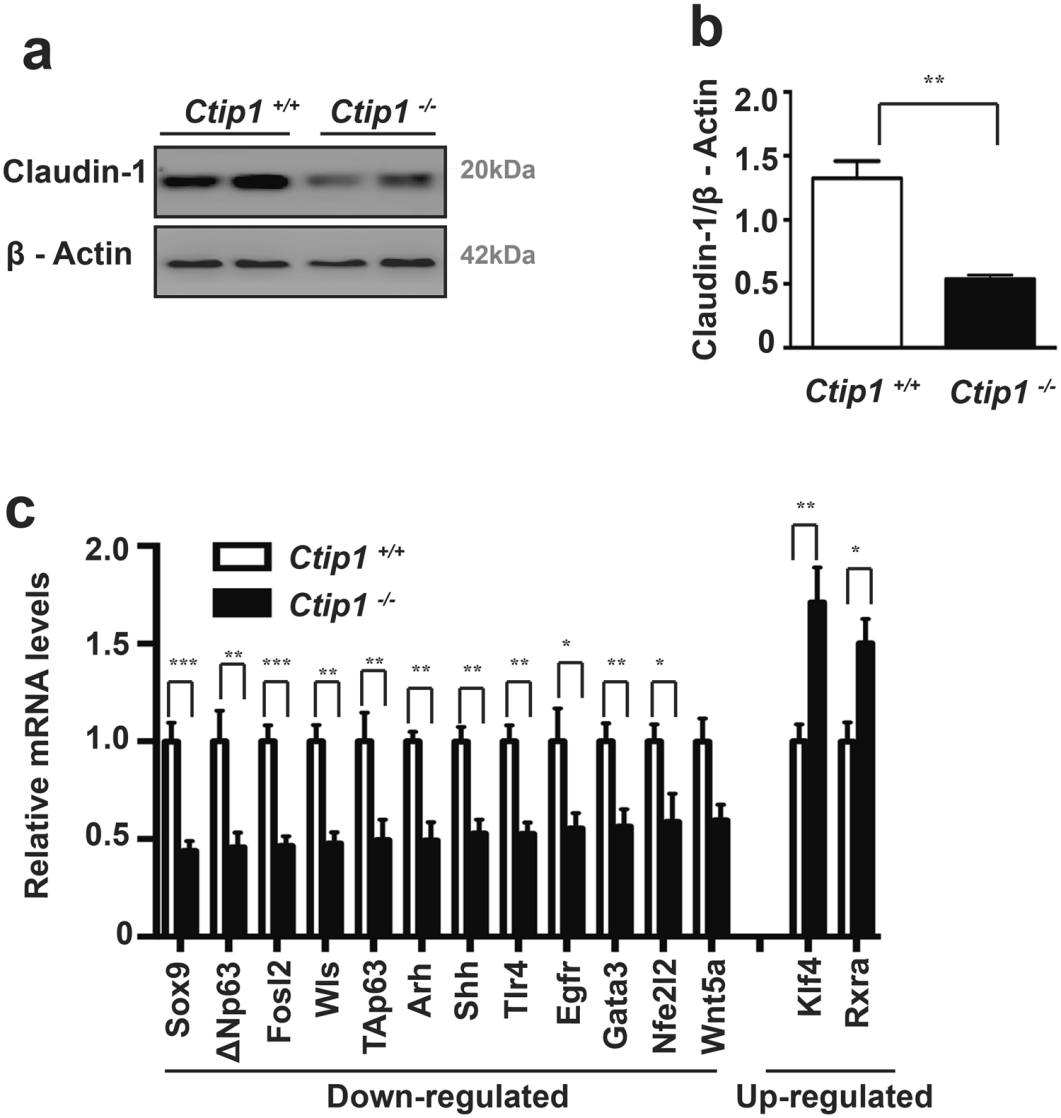
Altered expression of genes encoding transcription factors and intercellular junctional proteins in *Ctip1* mutant embryonic skin. (**a**) Immunoblot analysis for Claudin-1 on ventral skin extracts from E18.5 *Ctip1* ^+/+^ and *Ctip1* ^−/−^ embryos using antibody against Claudin-1. β-actin was used as a loading control. Full-length blots are presented in Supplementary Figure S7. (**b**) Quantification of Claudin-1 expression in the E18.5 *Ctip1* ^+/+^ and *Ctip1* ^−/−^ embryonic skin. (**c**) qRT-PCR validation of expression of several genes encoding transcription factors that were significantly altered in *Ctip1* ^−/−^ embryonic skin via RNA-seq (Figure S5d). Data are expressed as mean ± SEM (n = 8 biological replicates), *P < 0.05, **P < 0.01, ***P < 0.001. All qRT-PCR experiments were repeated three times.

We investigated expression patterns of genes encoding JPs, cytokines and chemokines, and antimicrobial peptides (AMPs), all of which are involved in barrier functions. RNA-seq analysis revealed that a subset of genes encoding JPs was differentially expressed in *Ctip1* ^−/−^ skin. We found that Claudin-1 (*Cldn1*), Claudin-2 (*Cldn12*) and Junctional Adhesion molecule-2 (*Jam2*) were down-regulated, while Claudin-15 (*Cldn15*), Tight junction protein-3 (*tjp3*), and Platelet and endothelial cell adhesion molecule-1 (*pecam1*) were up-regulated (Figure S5c). Among all of the junctional genes, *Cldn1* was most abundantly expressed in the WT skin and its expression was most significantly down-regulated in the mutants. Immunoblot analysis further confirmed the down-regulation of CLDN1 in mutant skin (Fig. 6a). In contrast, the expression profile of AMPs, cytokines and chemokines was not significantly altered in *Ctip1* ^−/−^ embryos (Tables S1, S2).

Several TFs regulate keratinocyte differentiation program, lipid metabolism, and intercellular junctions during formation of a functional skin barrier^14,18–20,35,38–40^. The expression of a subset of genes encoding these skin-related TFs was significantly altered in *Ctip1* ^−/−^ embryonic skin (Figs 6c and S5d). These genes included SPY-box containing gene 9 (*Sox9*), nuclear factor (*Nfe2l2*), *p63*, *Fosl2*, epidermal growth factor receptor (*Egfr*), wntless homolog (*Wls*), sonic hedgehog (*shh*), aryl-hydrocarbon receptor (*Ahr*), GATA binding protein 3 (*Gata3*), retinoid X receptor alpha (*RxRа*), and kruppel-like factor 4 (*Klf4*) (Fig. 6c). Among those significantly altered genes encoding TFs, ~85% of genes were down-regulated, while ~15% of genes were up-regulated (Figures S5d). These results suggest that CTIP1 might act as a key player to regulate expression of a subset of genes encoding TFs and proteins involved in intercellular junctions that are implicated in skin barrier formation.

### CTIP1 directly regulates the expression of *Fosl2, which is* involved in epidermal differentiation and *Elovl4* that is implicated in lipid metabolism

**Figure 7:**
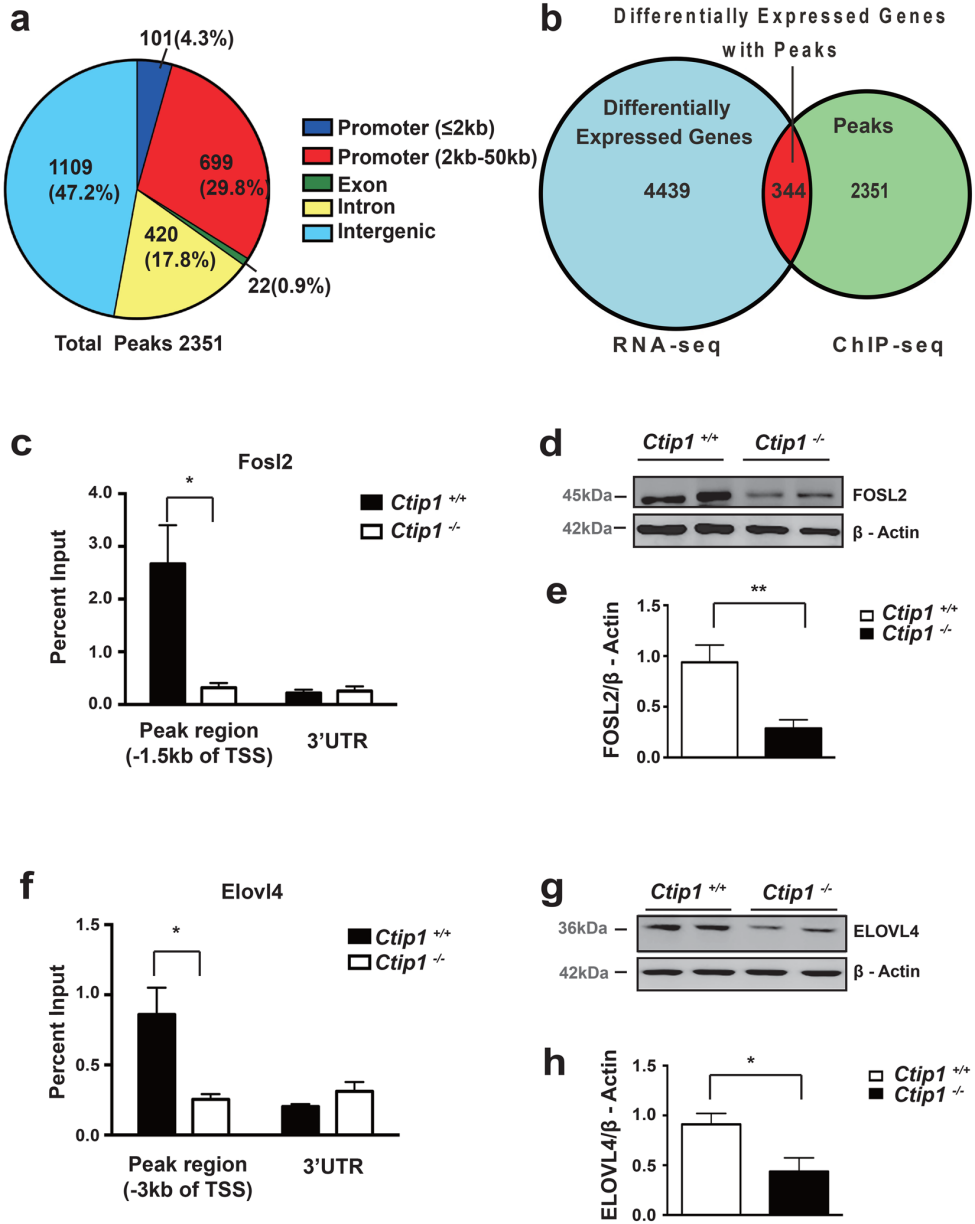
Identification and characterization of CTIP1 target genes in murine embryonic skin (**a**) Distribution of CTIP1 ChIP-seq peaks over proximal promoters (≤2 kb upstream of TSS), distal promoters (2–50kb), exons, introns, and intergenic regions. (**b**) Venn diagram represents the proportion of differentially expressed genes in *Ctip1* ^−/−^ embryonic skin (RNA-seq) with enrichment for CTIP1 peak by ChIP-seq. (**c**,**f**) ChIP-qPCR validation of ChIP-seq peaks at the ~1.5 kb upstream of TSS of Fosl2 (**c**) and ~3 kb upstream of TSS of Elovl4 (**f**). (**d**,**g**) Immunoblot of ventral skin extracts from *Ctip1* ^+/+^ and *Ctip1* ^−/−^ embryos at E18.5 using antibodies against FOSL2 (**d**) and ELOVL4 (**g**). β-actin was used as a loading control. Full-length blots are presented in Supplementary Figure S7. (**e**,**h**) Quantification of Fosl2 (**e**) and ELOVL4 (**h**) immunoblots, *P < 0.05, **P < 0.01.

To determine the mechanisms of CTIP1-mediated regulation of gene expression in skin, ChIP-seq analysis was performed on primary mouse skin keratinocytes using an anti-CTIP1 antibody. The specificity of the CTIP1 antibody was first confirmed by immunoblot (Figure S5e). Using the peak calling program MACS14, we identified 2351 statistically significant peaks in the E18.5 skin. The genomic distribution of peaks revealed that 34% of these peaks were located in gene promoter regions, with 4.3% in the proximal region [within 2kb upstream of transcriptional start site (TSS)] and 29.7% in the distal region (2kb–50kb upstream of the TSS). The remainder of the peaks were located in exonic (0.9%), intronic (17.9%), and the intergenic (47.2%) regions (Fig. 7a). Next, we integrated the ChIP-seq data with RNA-seq data to determine if the peaks were near genes that were significantly altered. We discovered that 344 differentially expressed genes (DEGs) had associated CTIP1 peaks in promoter, exon, intron, or intergenic regions (Fig. 7b). We performed enrichment analysis to determine which Gene Ontology (GO) terms in the biological process domain were associated with genes in this list more often than would be expected by chance. We found that “development process”, “metabolic process”, and “organic substance metabolic process” were among the top 10 significantly enriched GO biological process terms when the results were sorted by p-value (Table S3), while “cell development” and “cell differentiation” were among the top 25 if the results were arranged based on fold enrichment (Table S4). When we restricted the possible terms to the GO-Slim biological process set, we identified a single significant term “metabolic process”, which includes lipid metabolism. We then narrowed our focus to two genes that were associated with these enriched GO terms and have well defined roles in skin epidermal barrier differentiation and lipid metabolism, *Fosl2 and Elovl4* ^18,21,41,42^. FOSL2 is a key transcription regulator involved in the terminal differentiation of keratinocytes, and ELOVL4 is a fatty acid elongase involved in the synthesis of epidermis-specific long-chain and very long-chain CERs, which are essential components of the SC lipid barrier^41,43^. We next performed ChIP-qPCR assay to validate the ChIP-seq data. Our results confirmed that the promoter region of *Fosl2* (~1.5kb upstream of the TSS) and *Elovl4* (~3kb upstream of the TSS), had significant enrichment for CTIP1 binding over the IgG control (Fig. 7c,f). Interestingly, clusters of CTIP1 binding motif GGCCGG were observed at this position within the *Fosl2* (Figure S5f) locus^25^. Mapping of ChIP-seq data for CTIP1 and histone activation mark H3K4me3 revealed that the CTIP1 binding site on *Fosl2* (Figure S5f) and *Elovl4* (Figure S5g) was close to H3K4me3 modifications, which are usually found at active promoters. These observations were consistent with the reduction of *Fosl2 and Elovl4* expression in *Ctip1* ^−/−^ embryonic skin (Figs 5 b and 6c), suggesting that CTIP1 preferentially binds to active promoters and positively regulates the expression of these genes. The reduction of FOSL2 and ELOVL4 in mutant embryo was further confirmed at the protein level by immunoblot (Fig. 7d,e,g,h). These data suggest that *Fosl2* and *Elvol4* may be the direct targets of CTIP1, and play a role in mediating the effects of this TF on epidermal differentiation and lipid metabolism, respectively.

## Discussion

Skin epidermis serves as the first physical barrier to protect us from external assaults and water loss^4,44^. Transcriptional regulatory networks precisely orchestrate the establishment of functional skin barrier^2,18,22,35,45^. The present study reveals a previously unidentified role for the transcription factor CTIP1 in establishment and formation of epidermal permeability barrier during development. We have determined that CTIP1 is highly and continuously expressed in mouse skin epidermis during embryogenesis and in adulthood. Mice lacking CTIP1 displayed EPB defects accompanied by disrupted skin terminal differentiation, such as failed processing of pro-FLG into FLG monomers, and altered lipid metabolism. Skin barrier defects in *Ctip1* ^−/−^ embryos were likely due to abnormal expression of genes encoding skin lipid metabolizing enzymes, skin barrier-associated TFs and/or proteins that contribute to formation of tight junctions. Furthermore, we demonstrated that two of the newly identified target genes of CTIP1 in skin (*Fosl2* and *Elvol4*) are likely directly targets of this TF.

Impaired epidermal terminal differentiation, especially defective processing of profilaggrin, leads to the skin barrier defects^39,46,47^. Loss-of-function mutations in *FLG* gene have been identified as a cause of common skin diseases in rodents and humans characterized by disrupted EPB, such as AD and ichthyosis^48^. In *Ctip1* ^−/−^ embryos, we observed dramatic reduction of FLG monomers and a reduction in Caspase-14 expression, which processes pro-FLG to mature FLG and natural moisturizing factors^37^, This suggests a critical role of CTIP1 in FLG processing, perhaps through regulation of Caspase-14 expression. Additionally, the level of IVL, a maker of terminal differentiation^49^, was also significantly reduced in *Ctip1* ^−/−^ embryonic skin. These findings underscore the role of CTIP1 in epidermal terminal differentiation. The minimal change in keratinocyte proliferation in absence of CTIP1 can be explained in several ways. First, it is possible that CTIP1 mainly regulates epidermal differentiation, rather than proliferation. Indeed, we observed that some of the proliferating cells (Ki67^+^) in the epidermis did not co-express CTIP1 (Figure S1e). Second, it is possible that a putative, negative effect of CTIP1 on keratinocyte proliferation in *Ctip1* ^−/−^ embryos could be compensated by over-expression of other TFs, such as CTIP2 (Figure S2d). Generation of double-null mice (*Ctip1* ^−/−^|*Ctip2* ^−/−^) will be important to establish a functional redundancy between CTIP1 and CTIP2 in epidermal basal cells.

Epidermal terminal differentiation is a highly complex process and involves the finely balanced interplay of a handful of TFs^2,3,45^. A subset of terminal-differentiation-associated TFs such as *Fosl2, p63, Gata3, Ahr, Egfr, Shh and Sox9*, was dysregulated in the skin of *Ctip1* ^−/−^ mice. Individual knockout studies in mice for each of these genes have shown phenotypic and developmental abnormalities in the epidermis and hair follicles^19,21,23^. FOSL2, a member of the AP-1 family, is essential for epidermal barrier formation, especially in controlling terminal differentiation^21^. Skin barrier defects in *Fosl2* mutant mice are due to reduced expression of differentiation markers, such as FLG. This is similar to what we observed following ablation of *Ctip1*, which only affected epidermal differentiation. CTIP1 interacted with the promoter region of *Fosl2*, indicating that *Fosl2* might be a direct target of CTIP1. Furthermore, several GC-rich motifs (GGCCGG), to which CTIP1 binds directly^26^, are clustered around the promoter region of *Fosl2* and overlap the enriched CTIP1 peak as well a H3K4me3 mark. H3K4 tri-methylation is usually associated with promoters of transcriptionally active genes^50^. Although CTIP1 has been shown to act as a transcriptional repressor in erythroid cells^32,50^, our results indicate that CTIP1 might directly binds to the active promoter region of *Fosl2* gene through a GC-rich motif and positively regulate its expression. Therefore, the mechanism by which CTIP1 regulates gene expression could be context dependent and CTIP1 could act both as a repressor and/or an activator in a cell-specific manner, as it is true for most of the TFs.

In addition to proper execution of the keratinocyte terminal differentiation program, the presence of appropriate proportions of skin lipids is essential for protective skin barrier functions^12,51^. Alterations of skin lipid composition and organization lead to impaired skin barrier functions and result in an increase in TEWL that are seen in many skin diseases, such as AD, ichthyosis and psoriasis^52,53^. Loss of CTIP1 led to alterations in skin lipid composition, including alterations in ceramide composition and down-regulation of ceramide sphingoid base (e.g sphingosine and sphinganine) and ceramide precursor (e.g sphingomyelin). Consistent with the changes in lipid composition, Nile-red staining and ultrastructural analyses revealed abnormal surface lipid distribution and a reduction in the lipid content of the lamellar bodies in *Ctip1* ^−/−^ embryonic skin. LBs, formed in the stratum spinosum, are required for transfer and secretion of lipids and lipid-metabolism enzymes^54^. The *Ctip1* ^−/−^ skin showed decreased levels of ELVOL4, which is involved in the synthesis of very long-chain saturated or unsaturated fatty acids (≥C26) that are essential for long-chain ceramide synthesis^43^. Mice lacking this enzyme displayed EPB defects^41^. Indeed, a reduction in certain ceramide subclasses (e.g CER[NDS], CER[NS] and CER[EOH]) containing long-chain or very long-chain fatty acids was observed in *Ctip1* ^−/−^ embryos. The proportion of CERs with long-chain fatty acids was significantly lower in skin disease with barrier disruption^9,55^. Down-regulation of *Asah1* and *Sgpp1* along with the up-regulation of *Sphk1* and *Sphk2* in ceramide synthesis pathway may cause the observed decrease in sphingosine. The reduction in sphinganine might be due to decreased levels of *Kdsr*, part of the ceramide *de novo* synthesis pathway. The decreased level of ceramide sphingoid bases may further contribute to the overall reduction of CERs in the absence of CTIP1. ChIP-seq studies followed by ChIP-qPCR validation suggested a possible direct regulation of *Elovl4* by CTIP1 in skin. However, CTIP1 peak enrichment was not observed for other lipid metabolism genes that were differentially expressed in *Ctip1* ^−/−^ embryonic skin. So, CTIP1 might directly/indirectly regulate a subset of lipid-metabolism genes, and thus govern epidermal sphingolipids biosynthesis and metabolism.

Intercellular tight junctions, residing immediately below the stratum corneum, are required for protective barrier formation^15,56^. The Claudin-family of proteins is pivotal to tight junction formation. Deletion of *Cldn1* leads to skin barrier defects, alteration of ceramide composition and inhibition of FLG processing^57^. CLDN1 expression was decreased in AD skin characterized by impaired barrier function^58^. We observed changes in expression of junctional genes, especially *Cldn1*, in *Ctip1* ^−/−^ embryos. However, ChIP-seq analyses did not reveal binding of CTIP1 near the promoter regions of CLDN1, suggesting either CLDN1 may not be a direct target of CTIP1, or a more distal binding site is involved. In addition to the alteration of specific tight junction proteins, CDSN, a desmosome protein, was also changed in mutant skin. Loss of CDSN results in severe skin barrier dysfunction in mice and humans^59,60^. Therefore, alterations in junctional proteins in *Ctip1* ^−/−^ embryos may also contribute, in part, to the overall skin barrier defects we observed.

The intergration of ChIP-seq and RNA-seq data showed that only 8% of the differentially expressed genes in *Ctip1* ^−/−^ embryos skin had associated CTIP1 ChIP-seq peaks. It is possible that CTIP1 has multiple roles in the skin. In skin, CTIP1 could be directly regulating expression of a subset of genes, some of which might be transcription factors and directly regulating the expression of the rest. It is also possible that CTIP1 is a part of an interacting protein complex and serves as a cofactor and/or coregulator protein (co-activotor or co-expressor) that up- or down-regulated gene expression by binding to an activator or a repressor transcription factor, respectively, in a cell and context dependent manner.

The skin barrier abnormalities and lipid metabolism defects observed in *Ctip1* ^−/−^ embryos were reminiscent of the phenotype observed in *Ctip2* ^−/−^ embryos^14,35^. CTIP2 and CTIP1 are highly related genes, and both of which interact with COUP-TF family members^25^. Although co-expression of CTIP1 and CTIP2 in most of the basal cells was observed by IHC, some of the cells in the suprabasal epidermal layer were either CTIP1^+^ or CTIP2^+^ (Figure S2c), suggesting that those two proteins may have overlapping as well as unique functions in control of epidermal proliferation and differentiation. Our current study showed that CTIP1 had more of an effect on epidermal terminal differentiation, while our previous study revealed that CTIP2 controlled both epidermal proliferation and differentiation^35^. Both *Ctip2* ^−/−^ and *Ctip1* ^−/−^ embryos exhibit epidermal barrier defects, but each protein regulates distinct sets of skin- barrier related genes. Genes encoding sphingolipid-metabolizing enzymes (e.g *Kdsr, Asah1 and Sgms1*), which were altered in *Ctip1* mutant skin, were unaffected in *Ctip2* ^−/−^ embryos implying that *Ctip1* and *Ctip2* have distinct but overlapping roles in regulating epidermal barrier formation. Future research conducted with *Ctip1* ^−/−^ *| Ctip2* ^−/−^ double-knockout mouse would clarify those roles.

In conclusion, our data highlight importance of CTIP1 in the establishment of the EPB during development. The epidermal permeability barrier defects in *Ctip1* ^−/−^ embryos might be due to the combined effects of: (1) compromised terminal differentiation, such as disruption of filaggrin processing, (2) perturbations in lipid metabolism and altered lipid composition, and (3) alteration of epidermal junctional proteins, as summarized in Figure S6. Further studies in an epidermal-specific *Ctip1* knockout mouse model will be required to determine whether CTIP1 functions in a cell-autonomous or non-cell-autonomous manner for maintenance of skin barrier function and homeostasis. Our present study provides important knowledge regarding the transcriptional regulation of the EPB by CTIP1 and identifies new targets for treatment of skin diseases characterized by skin barrier disruption.

## Methods

### Generation of Mice

The CTIP1 mutant mouse line was established at the Phenomin - iCS (Phenomin - Institut Clinique de la Souris-, Illkirch, France; http://www.phenomin.fr). The targeting vector was constructed as follows. A 2.1 kb fragment encompassing exon 4 was amplified by PCR (from 129S2/SvPas ES cells genomic DNA) subcloned in an iCS proprietary vector. This vector contains a LoxP site as well as a floxed and flipped Neomycin resistance cassette. A 4.3 kb fragment corresponding to the 5′ homology arm and 2.8 kb fragment corresponding to the 3′ homology arms were amplified by PCR and subcloned in step1 plasmid to generate the final targeting construct. The linearized construct was electroporated in 129S2/SvPas mouse embryonic stem (ES) cells. After selection, targeted clones were identified by PCR using external primers and further confirmed by Southern blot with 5′ and 3′ external probes. Two positive ES clones were injected into C57BL/6J blastocysts, and male chimaeras derived gave germline transmission. Mice were housed in our approved University Animal facility with 12 h light cycles, food and water were provided ad libitum. All experiments were conducted in accordance with the relevant guidelines and regulations of the US, National Institutes of Health Guide for the Care and Use of Laboratory Animals. Oregon State University Institutional Animal Care and Use Committee (IACUC) granted institutional approval for all animal experiment.

### X-Gal diffusion assay

X-gal diffusion assay were performed as described previously^35^ with minor modifications. Freshly isolated embryos were washed with phosphate-buffered saline (PBS) for 5 min three times and then stained overnight at 37 °C in X-gal staining solution adjusted to pH 4.5. After staining, embryos were rinsed in PBS and fixed in PFA and photographed.

### Immunohistochemistry

Immunohistochemistry (IHC) staining of paraffin sections was described previously^61^. In brief, paraffin sections were deparaffinized in Xylene and rehydrated through graded alcohols. Antigen retrieval was performed with pH 6.0 cirtrate buffer (95–100 °C) for 20 minutes. Slides were washed with 0.1% PBS-Tween (PBST) and blocked with 10% Normal Goat Serum for 30 minutes. Sections were then incubated with primary antibodies overnight at 4 °C. Fluorescently labeled secondary antibodies were applied to slides for an hour at room temperature (RT). Antibodies used in IHC are detailed in Table S5. All sections were counterstained with DAPI for 10 min at RT to reveal nuclei. After the final washes, slides were dehydrated and mounted with DPX mounting medium. All images were captured using a Zeiss AXIO Imager.Z1 with a digital AxioCam HRm and processed using AxioVision 4.8 and Adobe Photoshop.

### Transepidermal water loss (TEWL) measurement

TEWL was assessed as previously described using a Tewameter TM300 with Multi Probe Adapter (CK electronic GmbH, Köln, Germany) in accordance with manufacture operating instruction^35^. Measurements were performed both at the dorsal and ventral skin site. Data was expressed in gm^−2^h^−1^, and presents the mean ± S.E.M from 6–8 independent animals (10 independent measurements per animal) of each genotype. Statistical analysis was performed using an unpaired Student’s t-test.

### Histological analysis

Skin biopsies were fixed in 4% paraformaldehyde overnight and embedded in paraffin blocks. 5 um paraffin sections were sectioned using Leica RM2255 microtome (Bannockburn, IL). Toludine-blue staining of 2 μm-thick skin sections and transmission-electron-microscopy analysis of 70 nm-ultra-thin sections were performed as described^62^.

### Nile Red staining

Frozen sections (10 μm) were stained with Nile Red and examined with Leitz fluorescence microscope (excitation 489 nm, emission 515 nm^35,62^).

### Immunoblotting analyses

Protein was extracted from mouse skin biopsies in a lysis buffer (20 mM HEPES, 250 mM NaCl, 2 mM EDTA, 1% SDS, 10% glycerol, 50 mM NaF, 0.1 mM hemin chloride, 5 mM NEM, 1 mM PMSF and protease inhibitor cocktail) followed by sonication. Protein concentrations were determined using the BCA assay (Thermo Scientific). Equal amounts of protein extract (10–20 μg) from each lysate were resolved using sodium dodecyl sulfate (SDS) polyacrylamide-gel electrophoresis and transferred onto a nitrocellulose membrane. Blots were blocked overnight with 5% nonfat dry milk and incubated with specific antibodies listed in Table S5 ^61^. The density of the band from the Immunoblotting was quantified using Rio-Rad Image Lab4.0 System (USA) and normalized by β-actin.

### Lipid extraction

Lipids from E18.5 embryonic skin were extracted according to the method of Bligh and Dyer with small modifications as described previously^14,63^. Briefly, skin epidermis was isolated from dermis by 1 mg/ml dispase. Skin epidermis was incubated in extraction solvent (chloroform:methanol:water(1:2:0.8), 10–15 mg tissue per 1 ml extraction solvent) O/N at room temperature. Fresh extraction solvent was added to epidermis samples on the second day and the old extraction solvent was saved. Samples were sonicated for 5 cycles, for 30 seconds each, and the suspension was shaken for 30 minutes at room temperature. After centrifugation at 2,000 r.p.m for 10 minutes, the pellets were discarded and the extraction solvents were combined. Chloroform and water were added to the extraction solvent at an extraction solvent:chloroform:water ratio of 7.6:2:2. After mixing and centrifuging the upper phase was discarded and the lower phase was washed twice with chloroform:methanol:water at a ratio of 1;1:0.9. The lower phase was dried in nitrogen, weighed and stored at −20 °C. The dry lipids were reconstituted in methylene chloride:isopropanol:methanol (23:10:65) before lipidomics analysis.

### Ultrahigh-pressure liquid chromatography/MS/MS (LC/MS/MS)

Ultra-pressure liquid chromatography was performed as previously reported on a Shimadzu Nexera system (Shimadzu, Columbia, MD) coupled with a quadrupole time-of-flight mass spectrometer (AB SCIEX, Triple TOF 5600) operated in information dependent MS/MS acquisition mode^63^. Data was imported into PeakView software for relative quantification and identification. Sphingolipids, CHOL and fatty acids species were confirmed by high resolution MS, MS/MS fragmentation, and isotopic distribution, and then compared using the PeakView database^63^. Sphingolipids, TAG and CHOL were identified in positive ion mode as [M+H]+ except ceramide as [M+H-H2O]+, and fatty acids in negative ion mode as [M−H]-, respectively.

### RNA extraction and RNA-seq

Total RNA was prepared from whole skin biopsies collected at E18.5 as described using TriZOL reagent (TriZOL Invitrogen) according to the manufacturer’s protocol^14^. RNA-seq was done by the center for Genome Research and Biocomputing (CGRB) core facility at Oregon State University (OSU). A sequence library was prepared using the Wafergen PrepX reagents. The RNA sequencing was done on an Illumina HiSeq. 2000 instrument, 100 bp paired-end using v.3. TruSeq cluster generation and SBS kits. For QC, the illumine libraries were checked with the Bioanalyzer HS-DNA chip. Then they were quantified by qPCR, using KAPA biosystems library quantification kit.

### Chromatin immunoprecipitation-sequencing (ChIP-seq)

ChIP-seq studies were performed on primary keratinocytes from newborn mice as previously described^23^. ChIP with 100 μg of chromatin solution was performed as described^64^ using specific antibodies against CTIP1 and a transcription activation mark [Histone 3, Lysine 4 tri-methylation (H3K4Me3)]. Construction of ChIP libraries and sequencing of 50 bp single–end reads on an Illumina HiSeq. 3000 were performed at the OSU CGRB core facility.

### RNA-seq and ChIP-seq Data analysis

The program Skewer was used to trim adaptors from reads and quality filtering was then performed^65^. Filtered reads were aligned to the mm10 genome^66^ using Hisat for RNA-seq data and Bowtie for ChIP-seq data^67,68^. The gene models came from Ensemble. Samtools was used for sam to bam file conversion and indexing^69^. For RNA-seq data analysis, the Cuffdiff program was used to estimate FPKM (fragments per kilobase of exon per million reads mapped) values for each replicates and to quantify differences in gene expression between the two experimental conditions (*Ctip1* ^−/−^ embryos and WT embryos)^70^. The Pearson correlation coefficient was calculated for all replicates (5 mutant and 5 WT) using SciPy^71^, and two WT replicates were discarded due to poor correlation with other replicates. For ChIP-seq data analysis, reads in regions known to have high artifact signal^72^ were removed using BEDTools^73^. A blacklist of high-artifact regions was obtained from https://sites.google.com/site/anshulkundaje/projects/blacklists. The peak-calling program, MACS14, was used to call peaks, the default seetings were used^74^. The command was: macs14 -t R2_ChIP_mm10_sort_blacklist.bam -c R2_ctl_mm10_sort_blacklist.bam -f BAM -g mm -n R2_macs. The UCSC Genome Browser^75,76^ (http://genome.ucsc.edu/) was used to visualize the mapping data and peak calls. A custom Python script was used to integrate the RNA-seq and ChIP-seq data^77,78^. Transcript and exon locations were extracted from an Ensembl release 75 GTF file^79^. The transcript loci and exons were compared against the location of the summit of all the peaks identified by MACS14. Transcripts with a TSS less than or equal to 2 kb downstream of the summit were classified as proximal, while those with a TSS between 2 kb and 50 kb downstream were classified as distal. If the summit was inside the transcript locus, the exons were checked to see if the summit was in an intron or exon. If the summit was not inside any transcript and there was no TSS within 50 kb, the peak was classified as intergenic. Once transcripts were identified, the RNAseq data produced by Cuffdiff was used to determine if peaks were near significantly expressed genes, and if so, whether those genes were up-regulated or down-regulated.

### qRT-PCR analysis

RNA was extracted and cDNA synthesis was performed using SuperScript III RT (Invitrogen) as described^14,61,62^. qRT-PCR amplification was performed on an ABI 7500 Real-Time PCR system using a SYBR Green methodology using specific primers as indicated in Table S6. *Hprt* was used as an internal control. All reactions were performed in triplicates.

#### ChIP-PCR analysis

ChIP assay was performed on primary keratinocytes from newborn mice according to Zhang *et al*.^23^. ChIP DNA was amplified using an ABI 7500 Real-Time PCR machine with specific primers indicated in Table S7. The results represent three separate experiments conducted in triplicate.

### Statistics analysis

All statistical significance of differences between different groups was assessed using GraphPad Prism software (Graphpad Software, La Jolla, CA) using Student’s unpaired *t*-test.

## Electronic supplementary material

Supplementary Information

Supplemental_TableS1-S4
