# Supplementary material for: Transcription Factor CTIP1/ BCL11A Regulates Epidermal Differentiation and Lipid Metabolism During Skin Development
Source: Sci Rep. 2017 Oct 18;7:13427. doi: 10.1038/s41598-017-13347-7 (PMC5647389; doi:10.1038/s41598-017-13347-7)
Supplement: Supplementary file 1 — Supplementary Information [file 41598_2017_13347_MOESM1_ESM.pdf]

Supplemental Information

Transcription Factor CTIP1/ BCL11A Regulates Epidermal Differentiation and Lipid Metabolism During Skin Development

Shan Li[1], Amy Teegarden[2], Emily M. Bauer[1], Jaewoo Choi [3], Nadia Messaddeq[4], David Hendrix[2], Gitali Ganguli-Indra[1,5], Mark Leid[1,2,5,6], Arup K. Indra[1,2,3,5,7,*]

[1]Department of Pharmaceutical Sciences, College of Pharmacy, OSU, Corvallis, Oregon, 97331, USA, [2]Department of Biochemistry and Biophysics, OSU, Corvallis, Oregon, 97331, USA, [3]Linus Pauling Science Center, OSU, Corvallis, Oregon, 97331, USA; [4]Institut de Génétique et de Biologie Moléculaire et Cellulaire, Department of Functional Genomics, Inserm U596 and CNRS UMR 7104, Illkirch, F-67400; [5]Knight Cancer Institute, Portland, Oregon, 97239, USA, [6]Department of Integrative Biosciences, Oregon Health & Science University (OHSU), Portland, Oregon, 97239, USA, [7]Departments of Dermatology, Oregon Health & Science University (OHSU), Portland, Oregon, 97239, USA

***Correspondence:** Arup K. Indra, E-mail: arup.indra@oregonstate.edu

Supplemental Figure Legends:

Figure S1. Expression of CTIP1 in the adult mouse

(a-b) IHC for CTIP1 (red) co-labeled with (a) basal keratinocytes marker K14 (green) and (b) suprabasal cell marker K10 (green) on skin sections collected from 8-weeks-old mice; (c) Western-blot analysis of protein extracts from E18.5 embryonic skin and 8-weeks-old mice skin using anti-CTIP1 antibody. β-actin was used as a loading control; (d) Quantification of immunoblot for CTIP1 expression; (e) Co-IHC staining for CTIP1 (red) and Ki67(green). While arrowheads indicate $CTIP1^{—}$| $Ki67^{+}$ cells and yellow basal cells are $CTIP1^{+}$| $Ki67^{+}$ cells. All sections are counterstained with DAPI (blue) to stain the nuclei. E, epidermis; D, dermis; HF, hair follicle; Scale bar = 50 μ m.

Figure S2. Characterization of *Ctip1* mutant embryos

(a) IHC analysis of CTIP1 localization in $Ctip1^{+/+}$ and $Ctip1^{-/-}$ embryonic skin at E18.5; (b) X-gal dye diffusion assay of $Ctip1^{+/+}$ and $Ctip1^{-/-}$ embryos at E17.5 and E18.5; (c) Co-IHC staining for CTIP1 (red) and CTIP2 (green) in E18.5 embryonic skin. Arrowheads indicate $CTIP1^{+}$| $CTIP2^{—}$ cells in the suprabasal layers; (d) Western-blot analysis of protein extracts from E18.5 $Ctip1^{+/+}$ and $Ctip1^{-/-}$ embryonic skin using anti-CTIP2 antibody. β-actin was used as a loading control. E, epidermis; D, dermis; HF, hair follicle; All sections are counterstained with DAPI (blue) to stain the nuclei. Scale bar = 50 μm.

Figure S3. Expression of proliferation markers and Caspase-14 in *Ctip1* mutant embryos

(a) IHC analysis of proliferation marker Ki67 in ventral skin of $Ctip1^{+/+}$ and $Ctip1^{-/-}$ embryonic skin at E18.5; (b) Graphical representation of percent $Ki67^{+}$ cells; (c) Immunoblot analysis using antibodies against PCNA was performed on $Ctip1^{+/+}$ and $Ctip1^{-/-}$ embryonic ventral skin at E18.5. β-actin was used as a loading control; (d) Quantification of immunoblot for PCNA expression. (e) qRT-PCR analysis of *Caspse-14* expression in E18.5 $Ctip1^{+/+}$ and $Ctip1^{-/-}$ skin. Data are expressed as mean ± SEM (n=8 biological replicates), * $P< 0.05$, * *$P< 0.01$. qRT-PCR for Caspase-14 was performed in triplicates.

Figure S4. Lipid profile of specific CER subclasses, saturated sphingomyelin, cholesterol, fatty acid and triglycerides in *Ctip1*$^{-/-}$ embryos

(a-c) Determination of (a) CER[NH], (b) CER[AH], and (c) CER[EOS] in the skin of E18.5 **of** *Ctip1*$^{+/+}$ and *Ctip1*$^{-/-}$ embryonic skin using LC-MS/MS. The X-axis defines the total carbon atom number while the Y-axis shows the absolute intensity (cps: count per second) of each lipid subclass. Each CER subclass is denoted by its sphingoid base and fatty acid chain. Sphingoid base abbreviations: H, 6-hydroxy sphingosine dihydrosphingosine; S, sphingosine. Acyl chain abbreviations: N, non-hydroxy fatty acid; A, hydroxyl fatty acid; EO, esterified ω-hydroxy fatty acid.; (d-g) Intensity of (d) saturated sphingomyelin, (e) cholesterol and cholesterol-3-sulfate; (f) saturated and unsaturated FFAs; and (g) saturated and unsaturated triglycerides by LC-MS/MS. Both FFAs and TGs are expressed as carbon number: double bonds number. Data are expressed as mean ± SEM (n=8), * *P*< 0.05, * **P*< 0.01.

Figure S5. Characterization of potential CTIP1 target genes by bioinformatics analyses

(a) Number of total differentially expressed, and up- or down-regulated genes; (b) Heat-map of expression of genes involved in sphingolipid metabolism. (c) Heat-map of gene expression for significantly altered junctional genes. (d) Heat-map of gene expression for significantly altered transcription factors involved in skin barrier functions. Each square represents the ratio of expression level of an individual sample/average wild-type expression. Red squares represent upregulation compared to the wild-type average, while blue squares show downregulation; (e) Determination of the specificity of the CTIP1 antibody by immunoblot. (f,g) Integrative UCSC genome tracks for (f) *Fosl2* and (g) *Elovl4*; H3K4me3 ChIP-seq data (violet); CTIP1 ChIP-seq data (the peaks identified by MACS 14 are denoted in orange); Input ChIP-seq data (grey).

Figure S6. Hypothetical model for CTIP1 functions in formation and establishment of epidermal permeability barrier (EPB)

CTIP1 controls formation and establishment of epidermal permeability barrier (EPB) through regulation of gene expression implicated in: 1) skin lipid composition, 2) epidermal differentiation, and 3) cellular junctions formation. Up- and down-regulated genes are indicated in red and blue, respectively. Potential CTIP1 target genes are marked by asterisks.

Figure S7. (a) The full-length blots of Figure 3b. (b) The full-length blots of Figure 3e. (c) The full-length blots of Figure 6b. (d) The full-length blots of Figure 7d. (e) The full-length blots of Figure 7g.

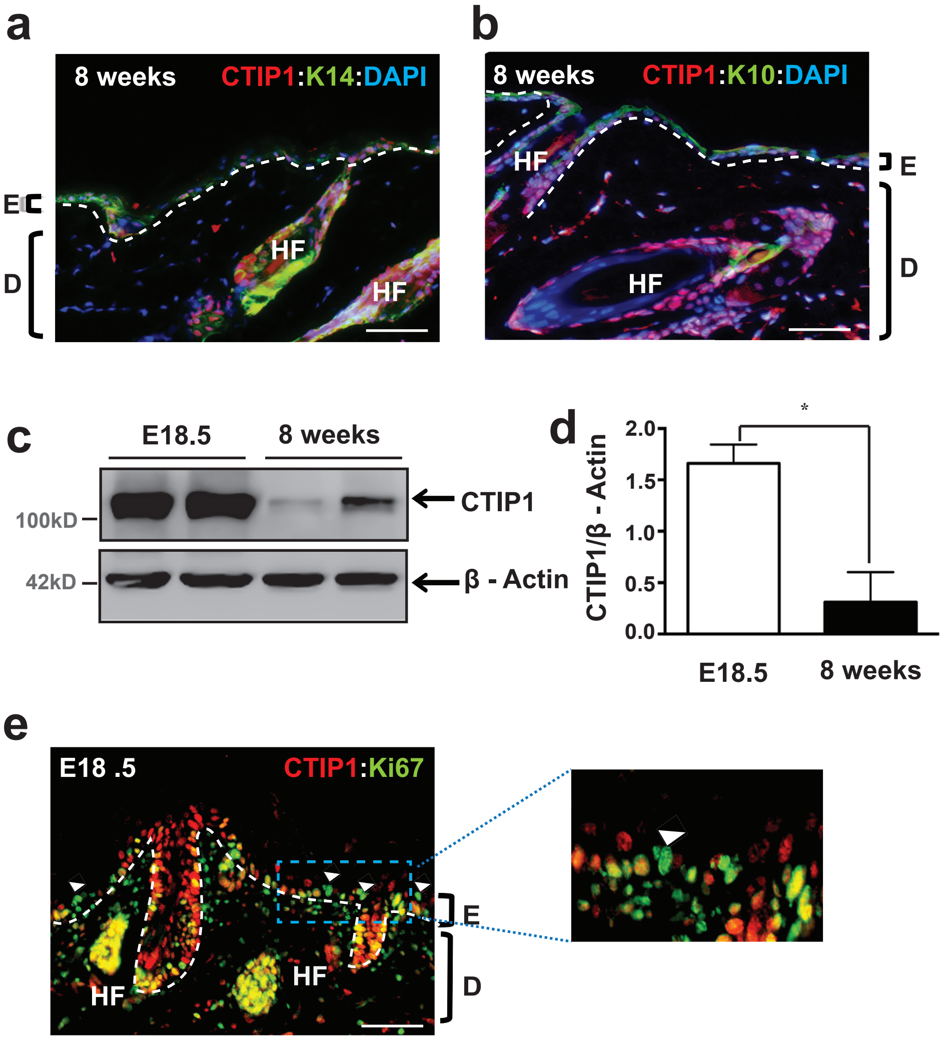

a
8 weeks
CTIP1:K14:DAPI
E
D
HF
HF
b
8 weeks
CTIP1:K10:DAPI
HF
HF
E
D
c
E18.5
8 weeks
100kD
42kD
CTIP1
β - Actin
d
CTIP1/β - Actin
2.0
1.5
1.0
0.5
0.0
*
E18.5
8 weeks
e
E18 .5
CTIP1:Ki67
E
D
HF
HF
HF

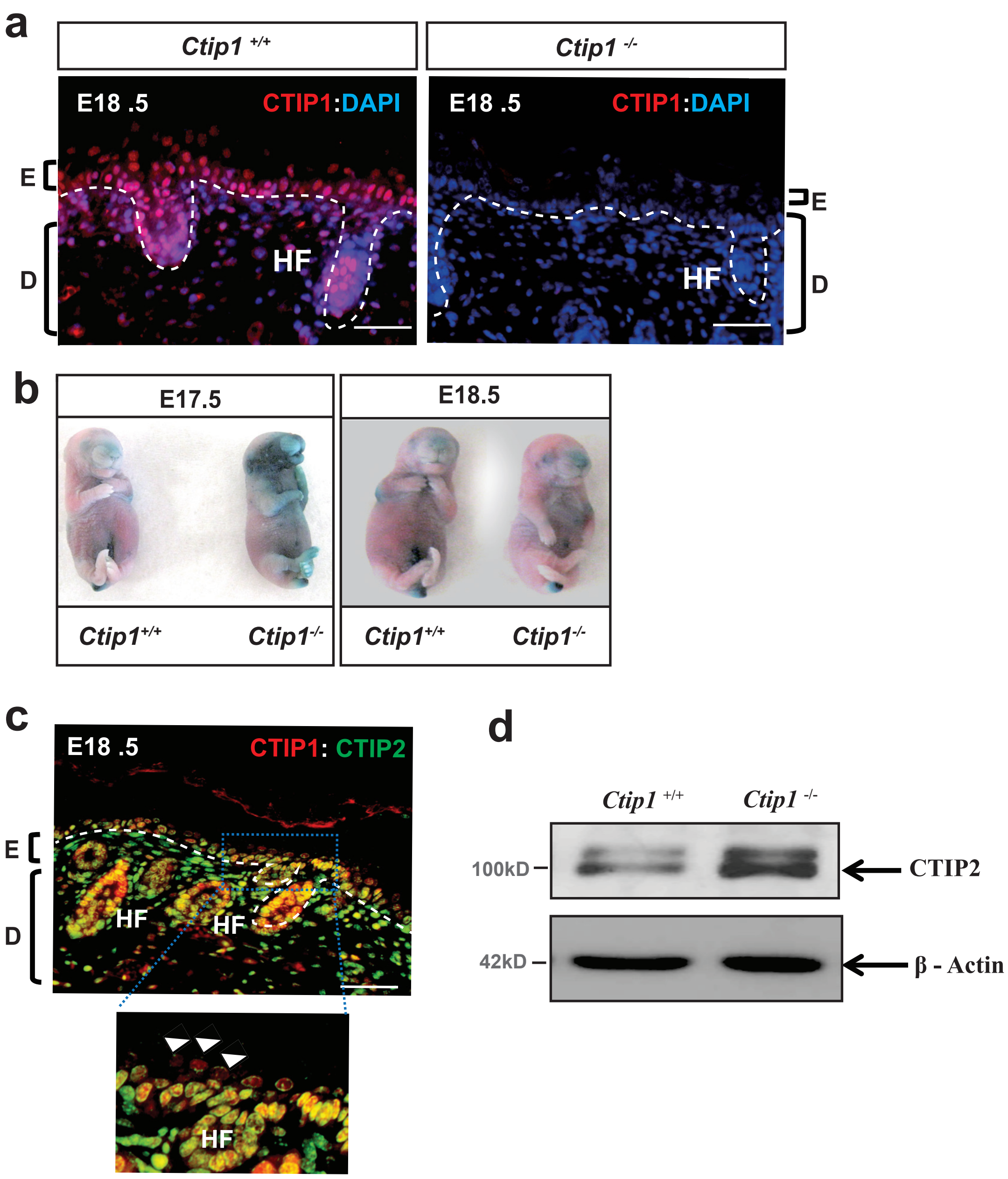
a
Ctip1 +/+
Ctip1 -/-
E18 .5
CTIP1:DAPI
E
D
HF
b
E17.5
E18.5
Ctip1+/+
Ctip1-/-
c
E18 .5
CTIP1: CTIP2
d
Ctip1 +/+
Ctip1 -/-
100kD
42kD
CTIP2
β - Actin

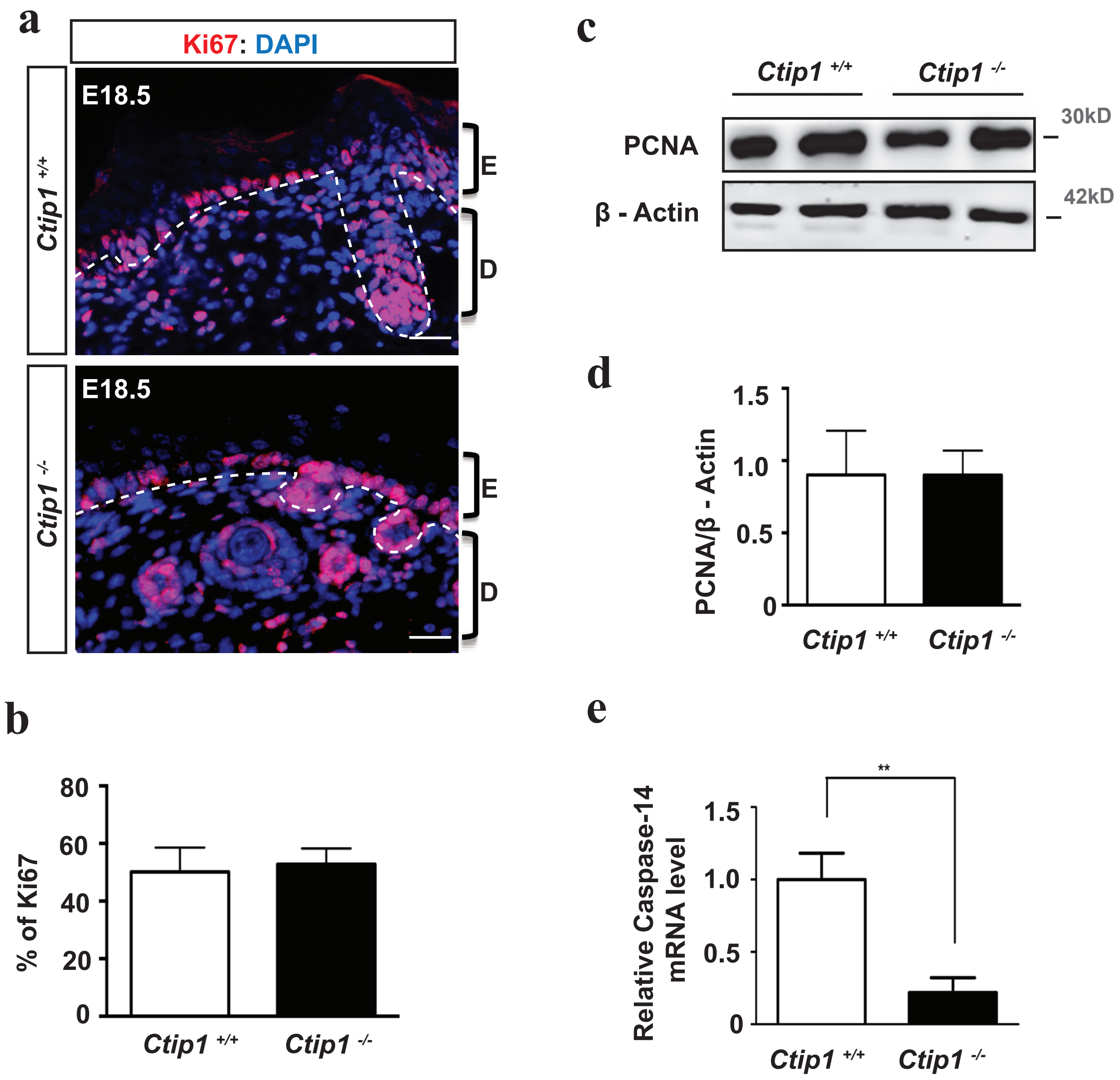
a
Ki67: DAPI
Ctip1 +/+
E18.5
E
D
Ctip1 -/-
E18.5
E
D
b
80
60
40
20
0
% of Ki67
Ctip1 +/+
Ctip1 -/-
c
Ctip1 +/+
Ctip1 -/-
PCNA
β - Actin
30kD
42kD
d
1.5
1.0
0.5
0
PCNA/β - Actin
Ctip1 +/+
Ctip1 -/-
e
**
1.5
1.0
0.5
0
Relative Caspase-14
mRNA level
Ctip1 +/+
Ctip1 -/-

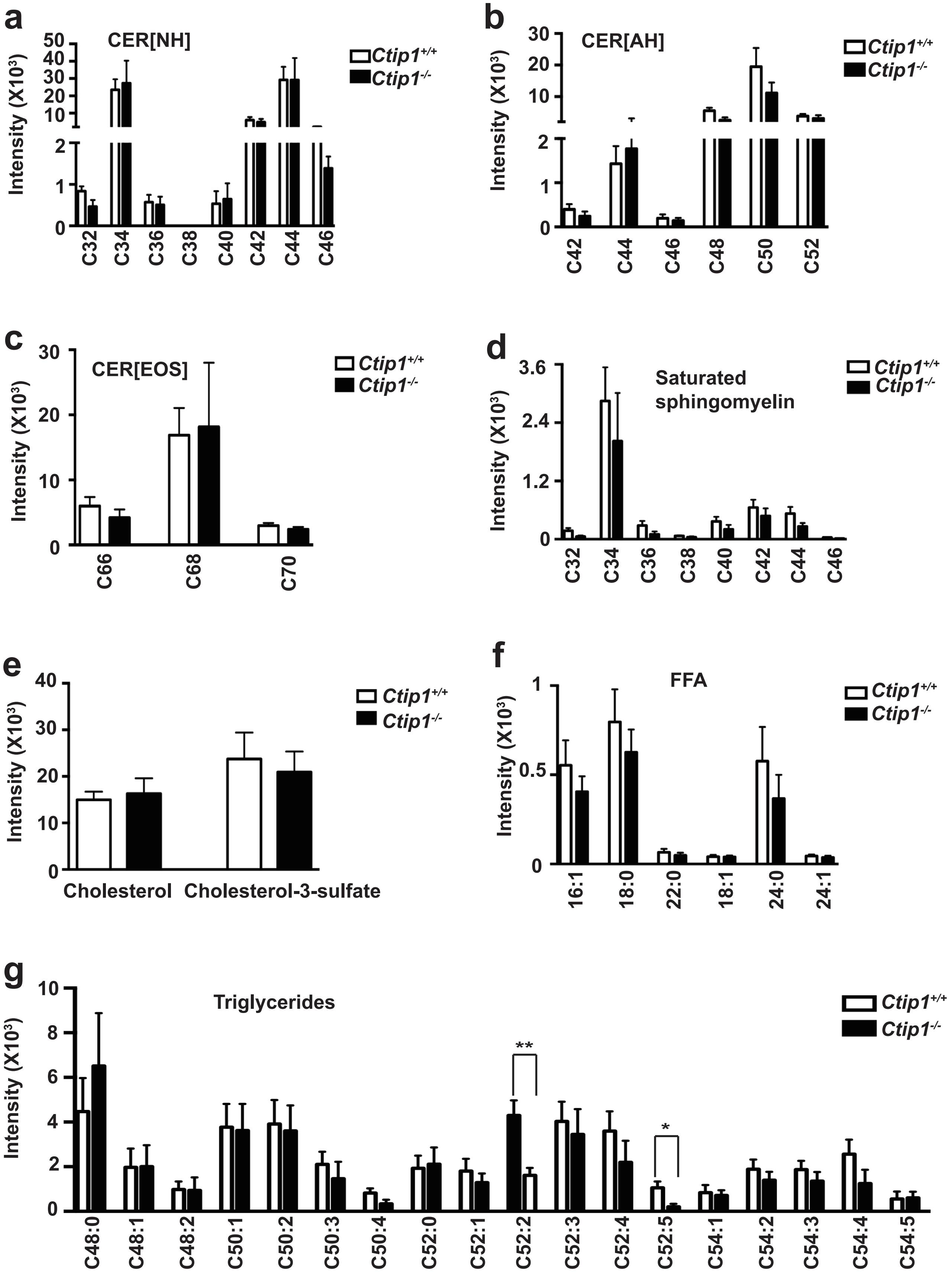
a
CER[NH]
Intensity (X10³)
Ctip1+/+
Ctip1-/-
C32
C34
C36
C38
C40
C42
C44
C46
b
CER[AH]
C42
C44
C46
C48
C50
C52
c
CER[EOS]
C66
C68
C70
d
Saturated sphingomyelin
e
Cholesterol
Cholesterol-3-sulfate
f
FFA
16:1
18:0
22:0
18:1
24:0
24:1
g
Triglycerides
C48:0
C48:1
C48:2
C50:1
C50:2
C50:3
C50:4
C52:0
C52:1
C52:2
C52:3
C52:4
C52:5
C54:1
C54:2
C54:3
C54:4
C54:5

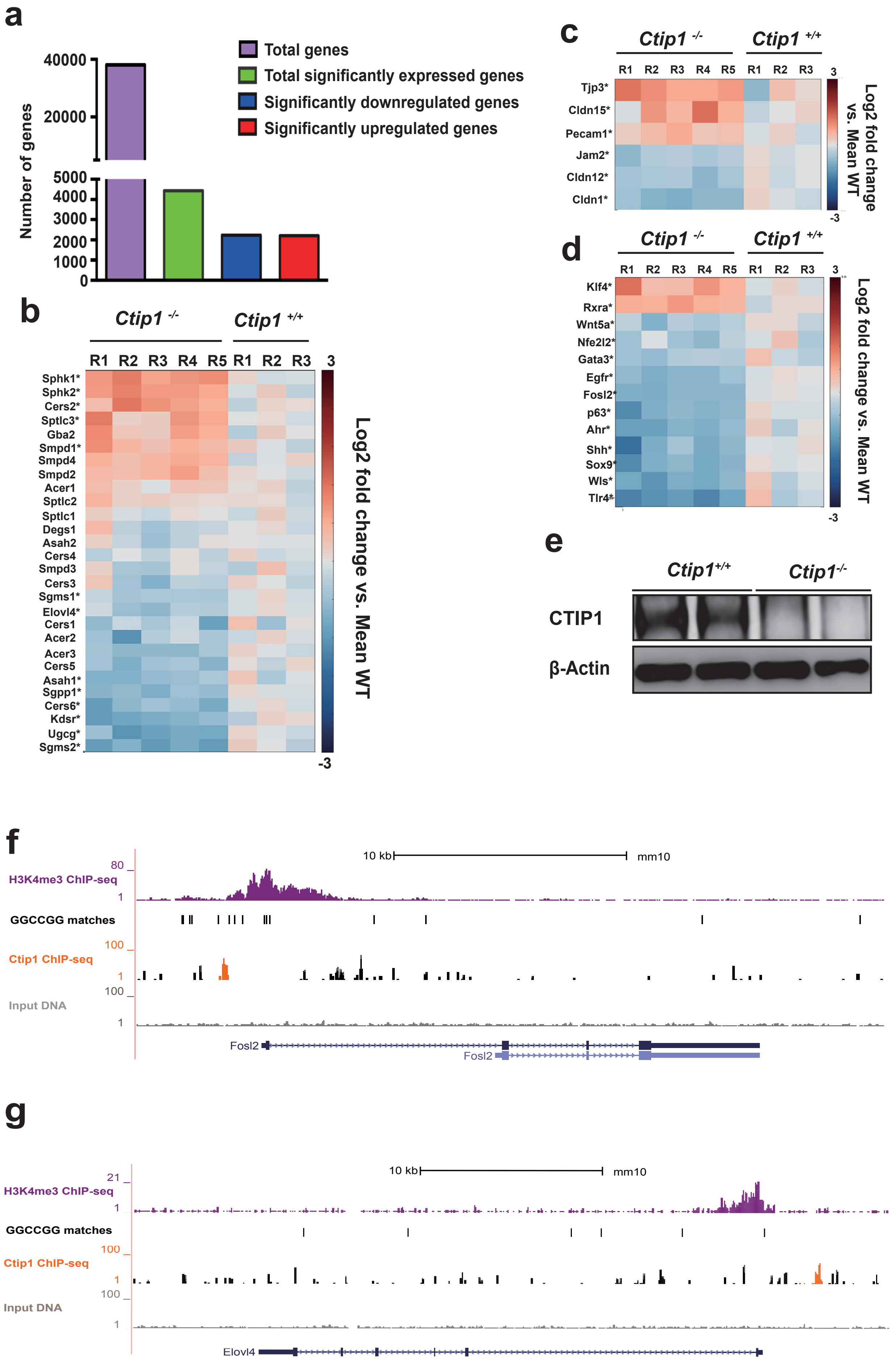

a
Total genes
Total significantly expressed genes
Significantly downregulated genes
Significantly upregulated genes
Number of genes
40000
20000
5000
4000
3000
2000
1000
0
b
Ctip1 -/-
Ctip1 +/+
R1 R2 R3 R4 R5 R1 R2 R3
Sphk1*
Sphk2*
Cers2*
Sptlc3*
Gba2
Smpd1*
Smpd4
Smpd2
Acer1
Sptlc2
Sptlc1
Degs1
Asah2
Cers4
Smpd3
Cers3
Sgms1*
Elovl4*
Cers1
Acer2
Acer3
Cers5
Asah1*
Sgpp1*
Cers6*
Kdsr*
Ugcg*
Sgms2*
3
-3
Log2 fold change vs. Mean WT
c
Ctip1 -/-
Ctip1 +/+
R1 R2 R3 R4 R5 R1 R2 R3
Tjp3*
Cldn15*
Pecam1*
Jam2*
Cldn12*
Cldn1*
3
-3
Log2 fold change vs. Mean WT
d
Ctip1 -/-
Ctip1 +/+
R1 R2 R3 R4 R5 R1 R2 R3
Klf4*
Rxra*
Wnt5a*
Nfe2l2*
Gata3*
Egfr*
Fosl2*
p63*
Ahr*
Shh*
Sox9*
Wls*
Tlr4*
3
-3
Log2 fold change vs. Mean WT
e
Ctip1+/+
Ctip1-/-
CTIP1
β-Actin
f
10 kb
mm10
80
H3K4me3 ChIP-seq
1
GGCCGG matches
100
Ctip1 ChIP-seq
1
100
Input DNA
1
Fosl2
Fosl2
g
10 kb
mm10
21
H3K4me3 ChIP-seq
1
GGCCGG matches
100
Ctip1 ChIP-seq
1
100
Input DNA
1
Elovl4

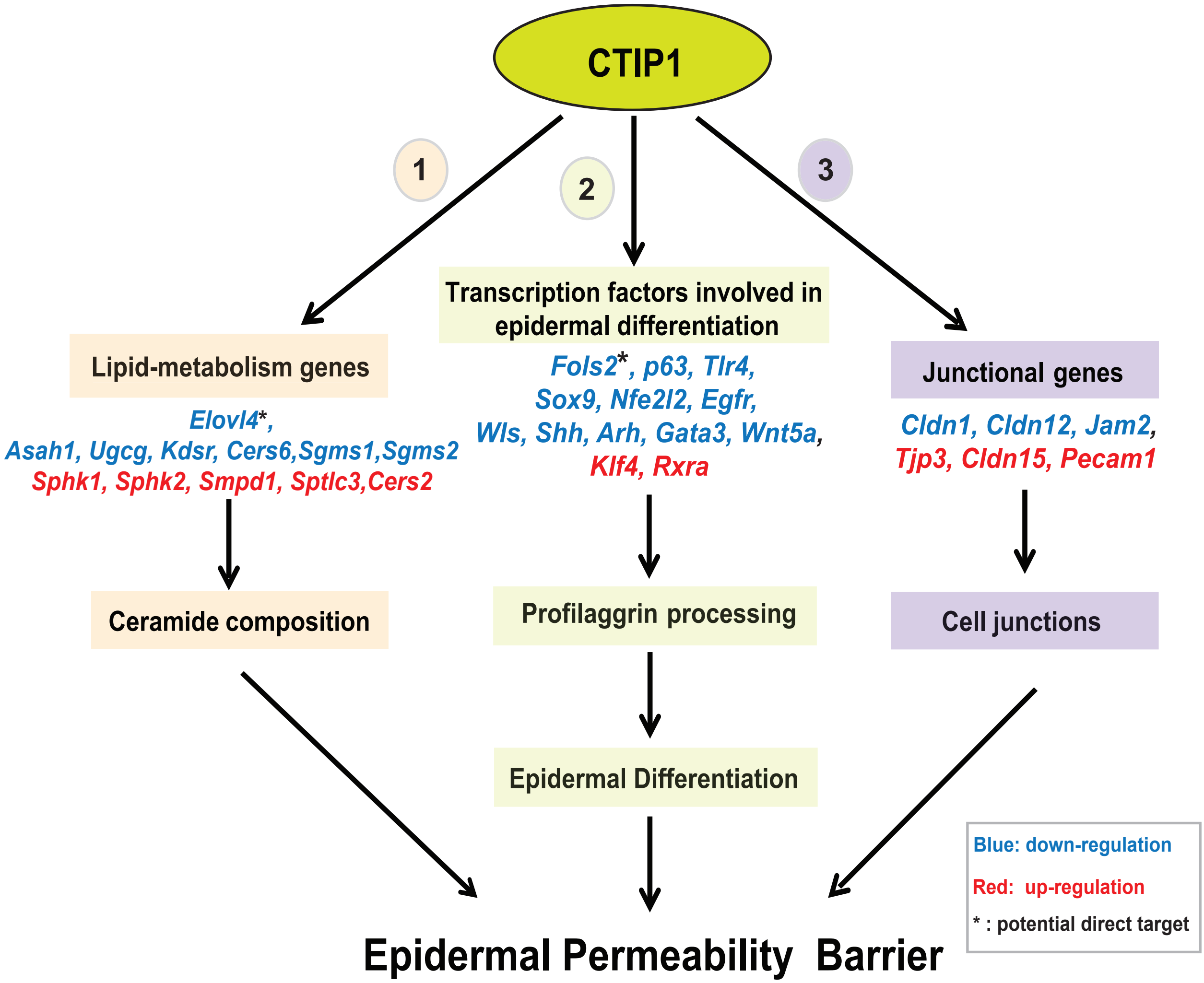

CTIP1
1
2
3
Lipid-metabolism genes
Elovl4*,
Asah1, Ugcg, Kdsr, Cers6,Sgms1,Sgms2
Sphk1, Sphk2, Smpd1, Sptlc3,Cers2
Transcription factors involved in epidermal differentiation
Fols2*, p63, Tlr4,
Sox9, Nfe2l2, Egfr,
Wls, Shh, Arh, Gata3, Wnt5a,
Klf4, Rxra
Junctional genes
Cldn1, Cldn12, Jam2,
Tjp3, Cldn15, Pecam1
Ceramide composition
Profilaggrin processing
Epidermal Differentiation
Cell junctions
Epidermal Permeability Barrier
Blue: down-regulation
Red: up-regulation
* : potential direct target

a. The full-length blots of Figure 3b.

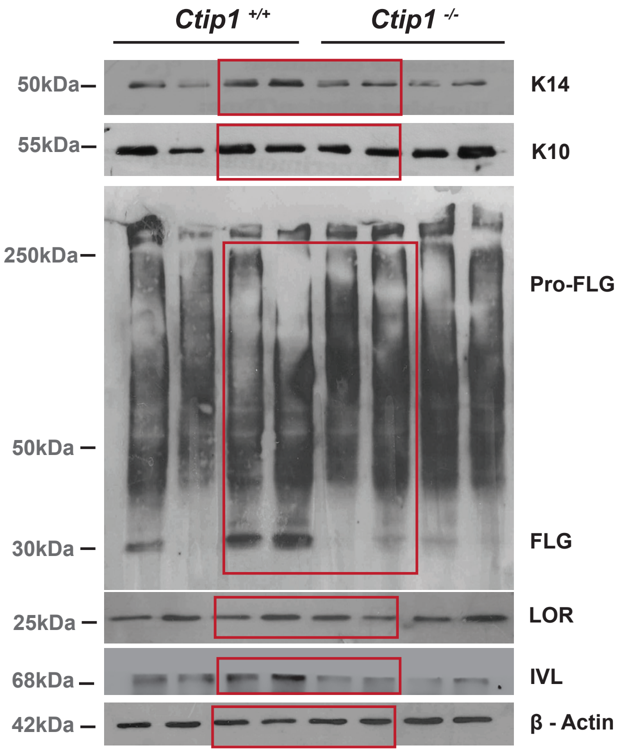

b. The full-length blots of Figure 3e.

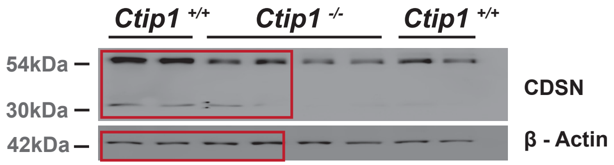

c. The full-length blots of Figure 6b.

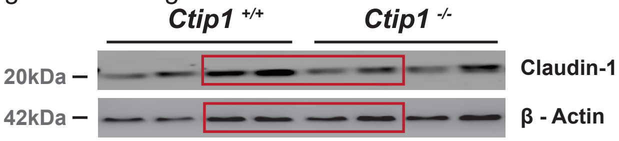

d. The full-length blots of Figure 7d.

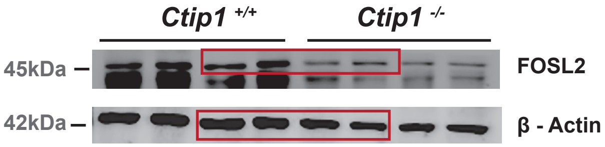

e. The full-length blots of Figure 7g.

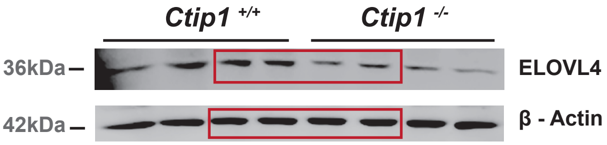

Table S5 List of Antibodies used for IHC and IB

Antibodies	Species	Source/Company	Dilution for IHC	Dilution for IB
anti – CTIP1	mouse	Abcam	1:200	1:1000
anti – K14	rabbit	Covance	1:1000	1:5000
anti – K10	rabbit	Covance	1:1000	1:5000
anti – β-actin	rabbit	Bethyl Laboratories		1:2000
anti –FLG	rabbit	Abcam	1:500	1:1000
anti –LOR	rabbit	Abcam	1:1000	1:1000
anti –INV	rabbit	Covance	1:1000	1:1000
anti-claudin-1 (Cldn1)	rabbit	Abcam		1:500
anti-CDSN	mouse	mAB F28-27		1:1000
anti - PCNA	mouse	Abcam		1:6000
anti – CTIP2	rat	Abcam	1:300	1:2000
anti – Ki67	rabbit	Abcam	1:500	
anti – Claudin1	rabbit	Abcam		1:1000
anti – FOSL2	rat	Millipore		1:1000
anti – ELOVL4	rabbit	Novus Biologicals		1:500
anti – TLR4	rabbit	Cell Signaling		1:1000
anti – p63	rabbit	Novus Biologicals		1:1000

Abbreviation used: Immunobloting (IB); Immunoflurescence (IF)

Table S6 List of primers used for RT-qPCR

Gene	Strand	Primer sequence
**Asah1**	forward	5’-TCCGTGGCACACCATAAATCT-3’
	reverse	5’-TCCACTTGGCACAAATGTATTCA-3’
**Ugcg**	forward	5’-TGCCTGGCATGGTTTATATT-3’
	reverse	5’-TAATGCCGACAGGAAAATGT-3’
**Kdsr**	forward	5’-CGGTGACTTCCATCACTGAA-3’
	reverse	5’-CTTCAGGTTTTGCTTTCTGC-3’
**Cers2**	forward	5’-TGATGGCAGTGCTACAGATG-3’
	reverse	5’-CCCCCTCTGAACTCTCTGTT-3’
**Cers6**	forward	5’-AAAAGGCAAGGTATCCAAGG-3’
	reverse	5’-CAAGGACCAGTGAGGAGGTA-3’
**Sgms1**	forward	5’-TCTGGTGGTATCACACGATG-3’
	reverse	5’-GCCAATGGTAAGATCGAGGT-3’
**Sgms2**	forward	5’-CTGGGATCATCTGCATTCTC-3’
	reverse	5’-GTTCGTCTGGGAAGAGACCT-3’
**Sphk1**	forward	5’-CACCAGAACGGAAGAACCAT-3’
	reverse	5’-GGTTTCTGGATGGCAGTCTC-3’
**Sphk2**	forward	5’-CTGCTTTACGAGGTGCTGAA-3’
	reverse	5’-CAACAGGTCAACACCGACAA-3’
**Smpd1**	forward	5’-TCACGTGGATGAGTTTGAGA-3’
	reverse	5’-CCGGGGTAGTTTCCATCTAT-3’
**Sptlc3**	forward	5’-CTACTTCCCTGCCAGAAGGT-3’
	reverse	5’-TATAGCACGCCCTGATTTCT-3’
**Elovl4**	forward	5’-CGCTCTATCTCCTGTTCGTGT -3’
	reverse	5’-GCGTTGTATGATCCCATGAAT-3’
**Sox9**	forward	5’-ATAAGTTCCCCGTGTGCATC-3’
	reverse	5’-TACTGGTCTGCCAGCTTCCT-3’
**Fosl2**	forward	5’- GCTCAGTGCCTTTTGGTTTC-3’
	reverse	5’- CTGGACCACCCAGATGAAAT-3’
**Nfe2l2**	forward	5’-CATGATGGACTTGGAGTTGC-3’
	reverse	5’-CCTCCAAAGGATGTCAATCAA-3’
**ΔNp63**	forward	5’-TTGTACCTGGAAAACAATG-3’
	reverse	5’-TCGAAGCTGTGTGGGCCCGGG-3’
**Egfr**	forward	5’-GCCATCTGGGCCAAAGATACC-3’
	reverse	5’-GTCTTCGCATGAATAGGCCAAT-3’
**Wls**	forward	5’-ATGGCTGGGGCAATTATAGAAAA-3’
	reverse	5’-GGGTGCTGGAGCGATCAAG-3’
**Shh**	forward	5’-AAAGCTGACCCCTTTAGCCTA-3’
	reverse	5’-TTCGGAGTTTCTTGTGATCTTCC-3’
**Arh**	forward	5’-CCCCGCTGAAGGAATTAAG-3’
	reverse	5’-AGCTCTTGGCCCTCAGGTAG-3’
**Gata3**	forward	5’-CCCCATTACCACCTATCCGC-3’
	reverse	5’-CCTCGACTTACATCCGAACCC-3’
**Klf4**	forward	5’-GTGCCCCGACTAACCGTTG-3’
	reverse	5’-GTCGTTGAACTCCTCGGTCT-3’
**Rxra**	forward	5’-ATGGACACCAAACATTTCCTGC-3’
	reverse	5’-CCAGTGGAGAGCCGATTCC-3’
**HPRT**	forward	5’-GTTAAGCAGTACAGCCCCAAA-3’
	reverse	5’-AGGGCATATCCAACAACAAACTT-3’

Table S7 List of primers used for ChIP-qPCR

Gene	Strand	Primer sequence
**Fosl2**	forward	5'- CGGGCCCCAGTTATTTATTT-3'
**(-1.3kb)**	reverse	5'- CCAGTGAGACATTCGGGAGT-3'
**Fosl2**	forward	5'-CAGTTTTGCTCCCTCTGGGT-3'
**(3'UTR)**	reverse	5'-GCTTCCCCAGCTCCAGAAAT-3'
**Elvol4**	forward	5'-GCCAAGCTGGAGAAAGGTAA-3'
**(-3kb)**	reverse	5'-CACATGCATTCTGGGTTCAA-3'
**Elovl4**	forward	5'-GGCCTGTTGAGGACAATGTT-3'
**(3'UTR)**	reverse	5'-CGTAAATCGTGGTGGCCTAT-3'
